# Improved tumor-type informed compared to tumor-informed mutation tracking for ctDNA detection and microscopic residual disease assessment in epithelial ovarian cancer

**DOI:** 10.1186/s13046-025-03433-4

**Published:** 2025-06-12

**Authors:** Mehdi Ben Sassi, Henri Azais, Charles Marcaillou, Sylvain Guibert, Emmanuel Martin, Jérôme Alexandre, Louise Benoit, Aurélien de Reynies, Emilie Laude, Cam Duong, Jacques Medioni, Bruno Borghese, Anne-Sophie Bats, Valerie Taly, Pierre Laurent-Puig

**Affiliations:** 1 Centre de Recherche des Cordeliers, INSERM UMRS1138, CNRS SNC 5096, Sorbonne Université, Université Paris Cité, Paris, France; 2 IntegraGen SA, Evry, France; 3 Department of Gynaecological Oncology and Breast Surgery, Assistance Publique-Hôpitaux de Paris, Hôpital Européen Georges Pompidou, Paris, France; 4 Institut du Cancer Paris Carpem, Assistance Publique-Hôpitaux de Paris, Paris, France; 5 Department of Medical Oncology, Assistance Publique-Hôpitaux de Paris, Hôpital Cochin, Paris, France; 6 Assistance Publique-Hôpitaux de Paris, Université Paris Cité, Hôpital Européen Georges Pompidou, Paris, France; 7 Department of Gynaecological Surgery, Assistance Publique-Hôpitaux de Paris, Site Cochin, Paris, France; 8 European Liquid Biopsy Society (ELBS), Hamburg, Germany; 9 Department of Genomic Medicine of Tumors and Cancer, Assistance Publique-Hôpitaux de Paris, Hôpital Européen Georges Pompidou, Paris, France

## Abstract

**Background:**

Epithelial ovarian cancer (EOC) is a leading cause of cancer mortality in women, often diagnosed at advanced stages. While first-line treatments improve survival, relapses remain common, with 5-year survival rates below 40%. Circulating tumor DNA (ctDNA) is a promising biomarker for non-invasive EOC detection and monitoring. It may help assess treatment response, notably microscopic residual disease. Our objective was to compare two ctDNA characterization strategies in EOC for assessing tumor burden during first-line treatment: a tumor-informed approach based on somatic mutations and a tumor-type informed approach utilizing DNA methylation patterns.

**Methods:**

In the tumor-informed approach, whole exome sequencing (WES) was performed on EOC tumor DNA and matched PBMCs from 22 patients to identify tumor-specific mutations. Personalized panels were then designed to track these mutations in plasma cfDNA. In the tumor-type informed approach, differentially methylated loci (DMLs) were identified by comparing EOC samples, healthy ovarian tissues, and PBMCs. A unique custom methylation panel was designed, and a support vector machine classifier was trained to distinguish between methylation profiles in plasma cfDNA from healthy donors and from EOC patients. Plasma samples from 47 advanced-stage EOC patients receiving chemotherapy and 54 healthy subjects were analyzed.

**Results:**

For the tumor-informed approach, WES identified an average of 72 somatic mutations per patient. For the tumor-type informed approach, 52,173 DMLs were identified as tumor-specific markers. In 47 plasma samples tested by both approaches, ctDNA levels were significantly correlated (*R* = 0.56, *p* = 4.3 × 10^-^^5^), with 70.2% concordance in detection. At baseline, ctDNA was detected in 21/22 patients with the tumor-informed approach, and in 11/12 non-training baseline samples with the tumor-type-informed classifier. At end-of-treatment, the latter detected ctDNA in 16/22 samples, outperforming the former. Detection using this more sensitive approach was significantly associated with relapse (log-rank *p* = 0.009; hazard ratio = 9.44; 95% CI 1.22–73.26) and poorer overall survival (log-rank *p* = 0.041).

**Conclusion:**

The tumor-type informed classifier demonstrated sensitivity and specificity for ctDNA detection, outperforming the tumor-informed approach in monitoring EOC progression. Requiring fewer sequencing data, it offers a practical, efficient solution for clinical management of EOC.

**Supplementary Information:**

The online version contains supplementary material available at 10.1186/s13046-025-03433-4.

## Introduction

Epithelial ovarian cancer (EOC) is the eighth most common cancer in women worldwide [1]. Due to unspecific symptoms, patients are often diagnosed at an advanced stage, characterized by a significant peritoneal tumor burden [2]. According to the International Federation of Gynecology and Obstetrics recommendations, the first-line treatment combines chemotherapy and cytoreductive surgery. Bevacizumab or Poly-ADP-ribose-polymerase inhibitors are commonly employed as maintenance treatments [3]. The absence of macroscopic residual disease after surgery remains the strongest prognostic factor for cure [4]. Despite first-line treatment and maintenance therapy, the 5-year survival rate remains below 40%, with frequent relapses [5]. Current follow-up methods, such as imaging and CA125 blood monitoring lack sensitivity, limiting early detection of recurrence and assessment of therapeutic response [6]. Indeed, assessing microscopic residual disease (MRD) after treatment could improve prognostication and therapeutic management of patients [7]. A reliable, non-invasive and sensitive monitoring approach of MRD is critically needed.

Analyzing circulating tumor DNA (ctDNA) shed by tumor cells into the bloodstream offers a promising non-invasive approach for both tumor burden assessment and characterization of tumor genomic alterations. In EOC, ctDNA analysis could enhance disease monitoring across multiple clinical scenarios, including screening, treatment response assessment, MRD evaluation after surgery and early detection of recurrence [8–10]. However, a major clinical challenge in EOC lies in the high heterogeneity of somatic mutations across patients [11]. Tumor-agnostic approaches eliminate the need for tumor tissue by leveraging knowledge of recurrent cancer alterations to develop standardized, one-size-fits-all assays [12]. These methods may involve broad genomic profiling, such as whole-exome sequencing (WES) and whole-genome sequencing (WGS) or focus on specific genes using PCR-based techniques such as digital PCR. While this approach offers advantages in terms of turnaround time and cost-effectiveness and has demonstrated high sensitivity for monitoring therapeutic response, it typically sacrifices sensitivity compared to tumor-informed approaches [12]. For instance, using a panel targeting nine commonly mutated genes in ovarian cancer, ctDNA was detected in only 69.2% of patient, leaving a significant proportion of cases undetected [13].

Tumor-informed approaches, by contrast, offer the highest sensitivity and specificity by tailoring the assay to each patient’s unique tumor profile. This strategy involves the identification of somatic mutations from the tumor tissue (via WES or WGS), followed by targeted monitoring in plasma [12]. To distinguish true somatic tumor variants from clonal hematopoiesis-related alterations, which are also present in cell-free DNA (cfDNA), sequencing of matched blood cells (e.g., PBMCs) is required [14]. In addition, the tumor-informed approach can reveal potential therapeutic targets or alterations associated with resistance mechanisms, thereby informing and optimizing treatment decisions [15].

In 2020, Zviran et al.. modeled ctDNA detection probability as a function of the number of genetic targets, cfDNA input and ctDNA fraction [16]. Their study demonstrated a clear advantage in increasing the number of monitored targets, showing that ctDNA fractions as low as 10^-^^5^ could be detected using tens of nanograms of cfDNA when up to 10,000 targets were analyzed. In EOC, the average tumor mutation burden is between 2 and 3 mutations per mega base, allowing for the identification of approximately 9,000 genomic alterations through WGS [17]. However, the cost and data burden of WGS limit its clinical applicability. WES, while more affordable, typically identifies only a few hundred mutations, thereby restricting the sensitivity of tumor-informed assays to ctDNA fractions of around 10^-^^4^.

To address this limitation, we sought to develop an intermediate tumor-type informed approach, which leverages epigenetic alterations recurrently observed across a specific cancer type [18]. By incorporating a sufficient number of such alterations, this strategy has the potential to achieve a sensitivity comparable to tumor-informed methods based on WGS identification of alteration, while retaining the versatility of a one-size-fits-all assay tailored to a given tumor type.

DNA methylation represents a promising avenue for ctDNA detection [19]. Recent advances have identified distinct methylation signatures across various cancer types, supporting the development of ctDNA-based detection strategies [20–23]. In this study, we applied a tumor-type informed approach in three key steps. First, we identified EOC-specific epigenetic markers using enzymatic conversion of unmethylated cytosines [24], followed by targeted enrichment, and next-generation sequencing (NGS). This enabled the identification of several thousand differentially methylated CpGs within a reduced genomic window, effectively distinguishing EOC tissue from healthy ovaries and PBMCs. Second, we evaluated the performance of these methylation markers in a series of plasma samples from EOC patients and compared the results to a tumor-informed approach based on WES of solid tumors and mutation tracking in plasma samples. Finally, we assessed the clinical potential of both approaches for detecting MRD following completion of first-line therapy.

## Materials & methods

### Sample collection

Patients included in this study were selected from the BIOVAIRE cohort (Paris, France). Of the 55 patients enrolled in the BIOVAIRE cohort, 47 were included in the present analysis. Eight were excluded due to the unavailability of baseline plasma samples. All participants provided informed consent, and the study was approved by relevant ethics committee (CPP n°: 2013-10-01 and ID-RCB: 2013-A01108-37). Tumor tissues were preserved in RNAlater and stored at -80 °C. Blood samples were collected in Streck tubes processed for plasma and PBMC isolation. V1 samples correspond to plasma collected prior to the initiation of chemotherapy. V2, V3, V4, V5, V6, and V7 refer to plasma samples collected after 1, 2, 3, 4, 5, or 6 cycles of chemotherapy, respectively. Chemotherapy cycles were administered every three weeks. V6 and V7 samples are considered end-of-treatment samples.

Normal ovarian tissues were obtained from OriGene (Herford, Germany) and classified as non-cancerous by two independent pathologists. Samples were flash-frozen in optimal cutting temperature (OCT) compound and stored at -80 °C. Publicly available whole-genome bisulfite sequencing (WGBS) datasets of normal ovarian tissue were downloaded from the ENCODE database (accession numbers: ENCSR417YFD and ENCSR803SIO). Blood samples from healthy female donors, collected in Streck tubes, were obtained from Biopredic International (UP, India) and l’Établissement Français du Sang (EFS, Évry, France). DNA from ovarian tissues and PBMCs was extracted using the Qiagen DNeasy Blood & Tissue Kit.

### Enzymatic-targeted methylation protocol and DMRs analysis

Libraries were prepared using the NEBNext Enzymatic Methyl-seq kit (New England Biolabs) with 100 ng of input DNA, followed by targeted hybrid capture using the Twist Human Methylome Panel (Twist Bioscience). To identify EOC-specific methylation markers, methylation profiles of ovarian tumors (*n* = 12) were compared to matched PBMC (*n* = 12) and normal ovarian tissues (*n* = 7). Sequencing was performed on an Illumina NovaSeq 6000 in paired-end mode (2 × 100 bp). Healthy ovarian controls tissues were enriched with two sets of FASTQ files downloaded from the ENCODE database. Sequencing reads were processed using Trim Galore (v0.6.6), BWAmeth (v0.2.7), Picard MarkDuplicates (v2.22.8), and methylation calling with MethylDackel (v0.6.0). CpG methylation profiles were compared between ovarian tumors and normal ovarian tissues, as well as between ovarian tumors and PBMCs from the same individuals, aiming to identify epigenetic markers highly specific to EOC. The sample sets used for marker selection ensure the panel’s relevance and specificity to the disease context by focusing on consistently hyper- or hypomethylated loci across multiple individuals. Differentially methylated loci (DMLs) were identified using DSS [25] and MethylKit R package [26], while differentially methylated regions (DMRs) were detected using DMRseq R package [27]. DMLs were defined using a methylation difference threshold of ≥ 30% and a false discovery rate (q-value) < 0.001, while DMRs were identified using the same methylation threshold and a significance cutoff of q-value < 0.01. CpGs identified as differentially methylated by at least two of the three methods were retained for panel design. Subsequently, DMRs were redefined by clustering the selected differentially methylated CpGs, requiring a minimum of four CpGs per region, each separated by less than 100 bp. Regions overlapping repetitive or low-complexity genomic elements were excluded from panel design.

### Targeted methylation sequencing of plasma samples

CfDNA was extracted from 3 mL of plasma using the QIAamp Circulating Nucleic Acid Kit (Qiagen). CfDNA quantification was performed using 1 µL of the 30 µL elution volume on a Fragment Analyzer (Agilent Technologies) with the NGS Fragment kit (1–6000 bp). Half of the remaining eluate (raging from 1.1 to 25 ng; mean 8.8 ng; IQR 3.5–10.6), was processed using the NEBNext Enzymatic-Methyl-seq kit with UMI methylated adapters (Twist Bioscience). CpG targets were selected based on the prior differential methylation analysis (see above), with repetitive and low complexity regions excluded to optimize enrichment efficiency. Based on this selection, a custom NGS capture panel was designed, comprising 24,199 baits of 120 bp each, covering a total genomic region of 2.79 Mb. Library hybridization was performed using this custom-designed panel according to the manufacturer’s recommendations (Twist Bioscience). Libraries were sequenced on an Illumina NovaSeq X system using 2 × 150 bp paired-end mode. To ensure uniform data across samples, sequencing output was normalized to 1.5 Gb per sample, resulting in an average raw sequencing depth of approximately 500x. Sequencing reads were aligned to the human reference genome (hg38) using BWAmeth (v0.2.7). Deduplication was performed using fgbio (v2.3.0) with UMI information to distinguish true duplicates from collision events (i.e., distinct fragments mapping to the same genomic coordinates). Quality control metrics such as mean on-target coverage, duplication rate, and enrichment efficiency were assessed for each sample to ensure robust and reproductible data acquisition. Methylation patterns at the resolution of individual reads [28] were identified using wgbstools [29]. A DNA fragment was considered tumor-derived if at least 85% of the DMLs within it matched the methylation status defined from tumor tissues.

For each DMR, a beta value was computed, representing the fraction of cfDNA fragments associated with a tumor-derived origin. To ensure specificity, DMRs with a standard deviation of beta values ≤ 0.05 across 24 healthy plasma donors were retained. The discriminative power of each DMR was assessed by computing the area under the receiver operating characteristic curve (AUC), comparing beta values from the 24 healthy controls and from 35 baseline plasma samples presumed to contain ctDNA. DMRs with an AUC ≥ 0.65 were selected for further analysis.

A Support Vector Machine (SVM) classifier was trained using the beta values from these selected DMRs as input features. The training set included the same 24 healthy and 35 baseline plasma samples used for DMRs selection. The trained model was then applied to classify additional plasma samples. Samples with a predicted value > 0 were classified as ctDNA-positive, while those with a value < 0 were considered ctDNA-negative.

### CtDNA proportion assessment using the tumor-type informed approach

To estimate the ctDNA fraction in plasma, we first refined our DMRs selection by retaining only those regions exhibiting extremely low background signal, defined as fewer than 1 in 10,000 reads displaying the tumor-associated methylation pattern, across the 54 healthy plasma donors. This filtering resulted in a final selection of 201 DMRs. For each ctDNA-positive sample, the ctDNA proportion was then estimated by computing the ratio of sequencing reads displaying the EOC-specific methylation signature to the total number of reads overlapping the coordinates of the 201 selected DMRs.

### DNA mixes for validation of ctDNA quantification using the tumor-type informed approach

To validate the ctDNA quantification strategy employed in the tumor-type informed approach, DNA mixes were prepared by combining DNA extracted from ovarian tumor tissues with matched PBMC-derived DNA at a ratio of 1:99. Tumor cellularity of the 12 ovarian tumor samples used for these mixes was determined based on copy number alteration (CNA) analysis performed on sequencing data generated for mutation profiling (see section below), using GATK (v4.1.4.1). The expected tumor DNA fraction in each mix was calculated as tumor cellularity divided by 100.

Each DNA mix was processed using the NEBNext Enzymatic Methyl-seq kit (Twist Bioscience), with 20 ng of fragmented input DNA and UMI-methylated adapters. Hybrid capture was performed using the custom panel targeting EOC-specific CpG sites, and sequencing was carried out on an Illumina NovaSeq X platform in 2 × 150 bp paired-end mode. Data analysis was conducted following the same pipeline as used for plasma samples.

### Identification of genetic alterations

**Fig. 1:**
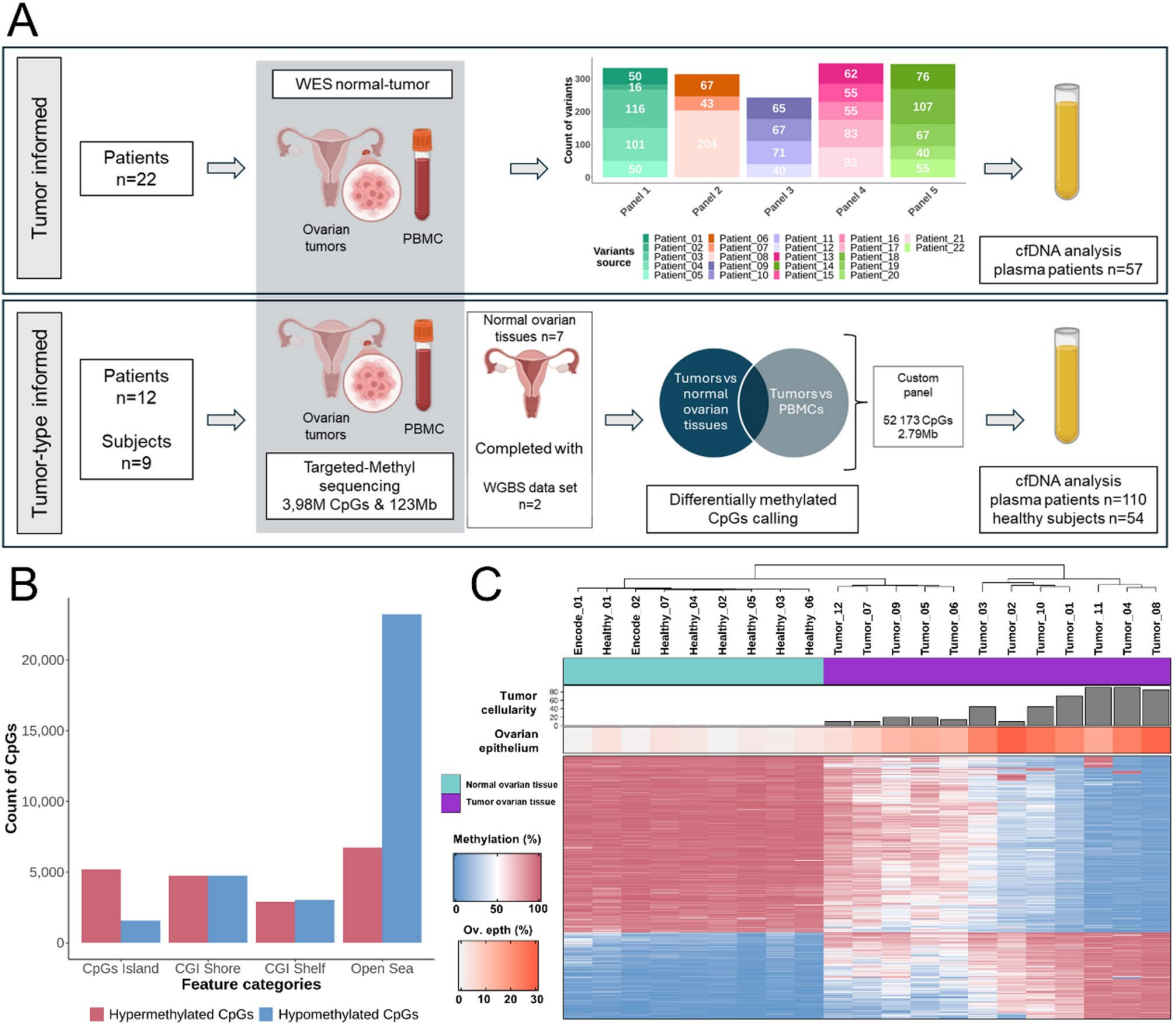
**A**. Overview of the study design. DNA was extracted from ovarian tumor tissues, matched patient PBMCs and normal ovarian tissues from independent donors. **Upper panel** illustrates the tumor-informed approach. Whole-exome sequencing (WES) was performed on tumor-PBMC pairs to identify tumor-specific SNVs. SNVs from tumors of 3–5 patients were grouped to design custom hybrid-capture panels, which were subsequently used for target-enrichment of plasma cfDNA libraries prior to sequencing. **The lower panel** shows the tumor-type informed approach: common tumors and PBMCs samples are used. These samples, along with normal ovarian tissues, underwent sequencing following the enzymatic-targeted methylation protocol. This protocol targets a 123 Mb genomic region, covering 3.98 million CpGs. Differentially methylated loci (DMLs) were identified by comparing the methylation profiles of ovarian cancer tissues to (i) healthy ovarian tissues, including two public whole-genome bisulfite sequencing (WGBS) dataset, and (ii) PBMCs. These identified DMLs were used to design a unique custom methylation panel, which was then applied to enrich cfDNA libraries from both patient and healthy donor plasma samples prior to sequencing. **B.** Genomic distribution of selected DMLs. DMLs are categorized as either hypomethylated (blue) or hypermethylated (red) in ovarian cancer tissues relative to both healthy ovarian tissues and PBMCs, and are distributed across CpG islands, shores, shelves, and open sea regions. **C.** Unsupervised clustering of ovarian samples based on the methylation levels of the 2,000 most variable DMLs. Top annotations indicate (from top to bottom): sample type (normal ovary, cyan; ovarian tumor, purple), tumor cellularity estimated from copy number alterations (based on WES), and the proportion of ovarian epithelial cells content inferred using the UXM_deconv algorithm [16] applied to the 3.98 million CpGs

Tumor and matched PBMC DNA from 22 patients underwent WES using the Twist Human Core Exome Panel (Twist Bioscience), with 100 ng of input DNA. Libraries were sequenced in paired-end 2 × 100 mode on an Illumina NovaSeq 6000 platform. Sequencing reads were aligned to the human reference genome (hg38) using BWA (v0.7.15), and PCR duplicates were removed with Sambamba [30]. Variant calling was performed with GATK HaplotypeCaller (v3.8.1) for germline DNA (PBMCs) and with MuTect2 (v2.0) for tumor DNA. Post-processing steps included artifact filtering and germline variant removal. All variants were annotated using the Variant Effect Predictor (VEP, v101). Based on these analyses, we selected for each patient, the somatic nucleotide variants that met the following criteria: an allelic frequency greater than 5%, a population frequency of the SNV considered to be below 0.5%, and a strong representation in the tumor, as reflected by both sequencing depth and allelic frequency. Each candidate SNVs was further assessed in the matched PBMC sample to exclude variants potentially associated with clonal hematopoiesis. We pooled the selected SNV from 3 to 5 patients to design 5 custom panels (Fig. 1A, top panel) using the Twist rapid MRD500 panel solution (Twist Bioscience). Pooling was performed to reduce sequencing costs while maximizing the number of patients analyzed per panel. Each custom panel was designed within the platform’s upper limit of 500 probes and covered a genomic region ranging from 40.3 to 47.3 kb.

### Detection of ctDNA in plasma based on the tumor-informed approach

Cell-free DNA from the remaining elution volume of plasma extractions (mean input: 9 ng; IQR 3.5–9.7 ng) was processed for mutation analysis using the Twist Mechanical Fragmentation Kit (Twist Bioscience). To enhance the detection of low-frequency tumor-specific SNVs, molecular barcodes were incorporated during library preparation, enabling the generation of consensus reads after sequencing. Libraries were target-enriched for tumor-specific SNVs genetic coordinates using associated designed panels. After targeted-enrichment, libraries were sequenced on an Illumina NovaSeq X using 2 × 150 bp paired-end mode. Sequencing reads were aligned to the human reference genome (hg38) using BWA (v2.2.1), and consensus reads were generated with fgbio (v2.3.0). Variant counting was performed using samtools mpileup (v1.9). To assess the reliability of ctDNA detection, the frequency of reads carrying tumor-specific SNVs was compared to the frequency of reads carrying unspecific SNVs at genomic positions confirmed to be non-mutated in the tumor and PBMC of each patient. For each tumor-SNV position, a p-value was computed using the binomial distribution 𝑃(𝑋=nb.tumor-SNV ∣ Depth, Freq.unspecific-SNV) where:

- nb.tumor-SNV is the number of reads carrying a tumor-specific SNV,
- Depth is the total reads depth at the SNV position,
- Freq.unspecific-SNV is the estimated background error rate, calculated per each base type and for each library from genomic regions confirmed to be non-mutated in both tumor and PBMC DNA of the patient (i.e. the distribution under null hypothesis *H*_*0*_).

Tumor-SNV with p-value ≥ 10^3^ were considered non-relevant. A sample was classified as ctDNA-positive when at least two tumor-specific SNVs were detected with p-values < 10^3^, corresponding to a combined false positive probability < 10^−6^. This statistical framework enables sensitive and reliable detection of ctDNA while accounting for background sequencing noise and sequencing errors specific to each sample and genomic context.

### ctDNA proportion assessment with the tumor-informed approach

ctDNA quantification in the tumor-informed approach was performed by computing for each ctDNA-positive sample, the ratio of sequencing reads harboring tumor-specific SNVs to the total number of reads overlapping the genomic coordinates of all tumor-identified SNVs in that patient.

### Statistical analysis

All statistical analyses were performed in the R environment (v4.0.2). The machine learning model was developed in Python (v3.7.2) with scikit-learn (v1.0.2), numpy (v1.21.6), pandas (v1.3.5) and seaborn (v0.12.2) packages. Deconvolution of methylation profiles to infer cellular origin was conducted using the UXM_deconvolution tool [31]. Progression-free survival (PFS) and overall survival (OS) were assessed based on ctDNA detection status. PFS was defined as the time from the end of first-line treatment to documented tumor recurrence. OS was defined as the time from initial diagnosis to death from any cause. Kaplan-Meier curves were generated to visualize differences in PFS and OS between patient groups, and a log-rank test was performed to compare the two groups. Hazard ratios (HR) were calculated using the survival R package. For OS, Firth’s correction was applied to the Cox regression model due to the absence of events in one of the groups, in order to reduce bias and improve model convergence.

## Results

### Characteristics of the study population

#### Patients

Baseline characteristics of the 47 patients included in this study are summarized in Table 1. The median age at diagnosis was 66 years (IQR: 60–71). All patients received standard-of-care treatment, including primary cytoreductive surgery followed by adjuvant chemotherapy (*n* = 23), neoadjuvant chemotherapy followed by interval cytoreductive surgery and adjuvant chemotherapy (*n* = 17), or chemotherapy alone (*n* = 7). The median follow-up duration for the 47 patients was 84 months (IQR: 35.8-not reached). Among these patients, 33 were analyzed at multiple time points, resulting in a total of 63 plasma samples collected during chemotherapy (V2-V7, Supplemental Fig. 1).

**Table 1:** Characteristics of the 47 study patients

	*N* = 47^*1*^
Age at diagnostic (median; IQR)	66; 60–71
BMI (median; IQR)	22.48; 20.8-27.66
Menopausal	41 (87%)
*BRCA* mutation	
Negative	37 (78.7%)
BRCA 1	2 (4.3%)
BRCA 2	2 (4.3%)
Not investigated	6 (12.7%)
ASA score	
1	10 (21.3%)
2	21 (44.7%)
3	7 (14.9%)
No data available	9 (19.1%)
Histology	
Clear cell adenocarcinoma	1 (2%)
Endometrioid carcinoma	2 (4.3%)
Low grade serous carcinoma	2 (4.3%)
High grade serous carcinoma	42 (89.4%)
FIGO stage	
FIGO II B	1 (2%)
FIGO III A	3 (6.5%)
FIGO III B	4 (8.5%)
FIGO III C	25 (53.2%)
FIGO IV	14 (29.8%)
Surgical resection	44 (88%)
CC0	38 (80.9%)
CC1	1 (2%)
CC2	1 (2%)
30-day postoperative mortality	2 (4.3%)
Therapeutic sequence	
Upfront surgery followed by adjuvant chemotherapy	23 (49%)
Neoadjuvant chemotherapy followed by interval surgery V3/V4/V5	16 (34%)
Neoadjuvant chemotherapy followed by surgery after V6	1 (2%)
Chemotherapy alone	7 (15%)
Total number of chemotherapy cycles (median; IQR)	6; 6–7
Follow-up in months (median; IQR)	84; 35.75-not reached
Recurrence rate	26 (55.3%)
Time from last chemotherapy to recurrence (median; IQR)	11.7; 9.43–16.1
Mortality rate	17 (36%)
Lost to follow-up	1 (2%)
Time from death to last chemotherapy (median; IQR)	24.2; 17.6–46.6
^*1*^ *median (iqr); n (%)*	

IQR: interquartile range; BMI: body mass index; ASA: physical status score; BRCA: breast cancer gene; FIGO: international federation of gynecology and obstetrics; CC: cytoreduction score

#### Healthy subjects

Plasma samples from healthy women were obtained from Biopredic International (median age 64 years; IQR 53–75) with no history of gynecological diseases. Additional plasma samples were provided by the Établissement Français du Sang (EFS), from women aged 40 to 50 years, also with no reported disease. Healthy ovarian tissue samples were acquired from Origene (*n* = 7; median donor age: 52 years; IQR: 45–59). To further expand the dataset, whole-genome bisulfite sequencing (WGBS) data from ENCODE database were incorporated, including ovarian tissue samples from two female donors aged 30 and 51 years.

### Characterizing genetic and epigenetic alterations in ovarian tumors, PBMC and healthy ovarian tissues for custom panels design

To evaluate and compare the performance of patient tumor-informed and tumor-type informed approaches for ctDNA detection and quantification, we characterized both genetic and epigenetic features of ovarian tumors. For the patient tumor-informed approach, WES was performed on DNA extracted from ovarian tumors and matched PBMCs from 22 patients from the BIOVAIRE cohort. We identified between 16 and 204 SNVs per patient (mean = 72; Fig. 1A, upper panel; see Methods for additional details).

Mutations in *TP53* were detected in 16 out of the 22 patients (73%), while mutations in *TTN* and *CSMD3* were observed in 6 (27%) and 5 (23%) patients, respectively. However, no recurrent hotspot mutations were identified.

For the tumor-type informed approach, we endeavored to identify specific methylation patterns of EOC using enzymatic conversion and library enrichment on a panel targeting 3.98 million CpGs by comparing tumor samples (*n* = 12) with healthy ovarian.

tissues (*n* = 7) and PBMCs (*n* = 12). PBMC samples were analyzed because the majority of cfDNA present in plasma samples originates from these cells. The two WGBS datasets were added to the healthy ovarian tissues group. Based on this approach, we identified 52,173 CpGs that were consistently differentially methylated in the same direction (either hypomethylated or hypermethylated) when comparing ovarian tumors to both healthy ovarian tissues and PBMCs (Fig. 1A, bottom panel). The distribution of DMLs across genomic regions revealed distinct patterns of hypermethylation and hypomethylation (Fig. 1B). In CpG islands, 5,179 CpGs were hypermethylated, compared to 1,574 that were hypomethylated, highlighting a pronounced bias toward hypermethylation in these regions. In contrast, open sea regions exhibited a predominant pattern of hypomethylation, with 23,239 CpGs hypomethylated versus 6,730 hypermethylated. In the shores and shelves of CpG islands, the distributions of hypermethylated and hypomethylated CpGs were balanced. Specifically, CpG island shores contained 4,756 hypermethylated and 4,751 hypomethylated CpGs, while shelves showed a slight bias toward hypomethylation, with 3,042 hypomethylated and 2,902 hypermethylated CpGs. These findings underscore the region-specific nature of DNA methylation alterations in ovarian tumors, with hypermethylation predominantly occurring in CpG islands and hypomethylation prevailing in open sea regions.

To evaluate the ability of the selected DMLs to discriminate ovarian tumor tissues from healthy ovarian samples, we performed unsupervised clustering based on the 2,000 DMLs showing the highest variability in methylation levels across all samples. This analysis revealed three distinct clusters, with healthy ovarian tissues grouping together. Notably, the separation between tumor and healthy samples increased with both tumor cellularity and the proportion of ovarian epithelial cells (Fig. 1C, Supplemental Fig. 2). These findings indicate that the selected DMLs effectively distinguish malignant from normal ovarian tissues and support their use in designing a targeted methylation panel spanning a 2.79 Mb genomic region.

### Tumor-informed approach for ctDNA characterization

After identifying tumor-specific SNVs for each patient and designing the custom panels, we analyzed 57 plasma samples collected from 22 patients at various time-points: baseline (V1, *n* = 22), during treatment (V2-V5, *n* = 20), and at the end of treatment (V6-V7, *n* = 15). Sequencing generated an average of 15.21 Gb of data per sample (range: 7.65–21.00 Gb). ctDNA presence in plasma samples was assessed by comparing the frequency of reads carrying tumor-specific SNVs to the background sequencing error rate for each nucleotide, which was determined at genomic positions without variations in both tumor and PBMC samples from the corresponding patients (Supplemental Fig. 3). This method provided a robust framework for evaluating ctDNA detection reliability in each sample. Among the 57 plasma samples, 42 were classified as ctDNA-positive, each containing at least two tumor-specific SNVs with a p-value < 10^− 3^.

**Fig. 2:**
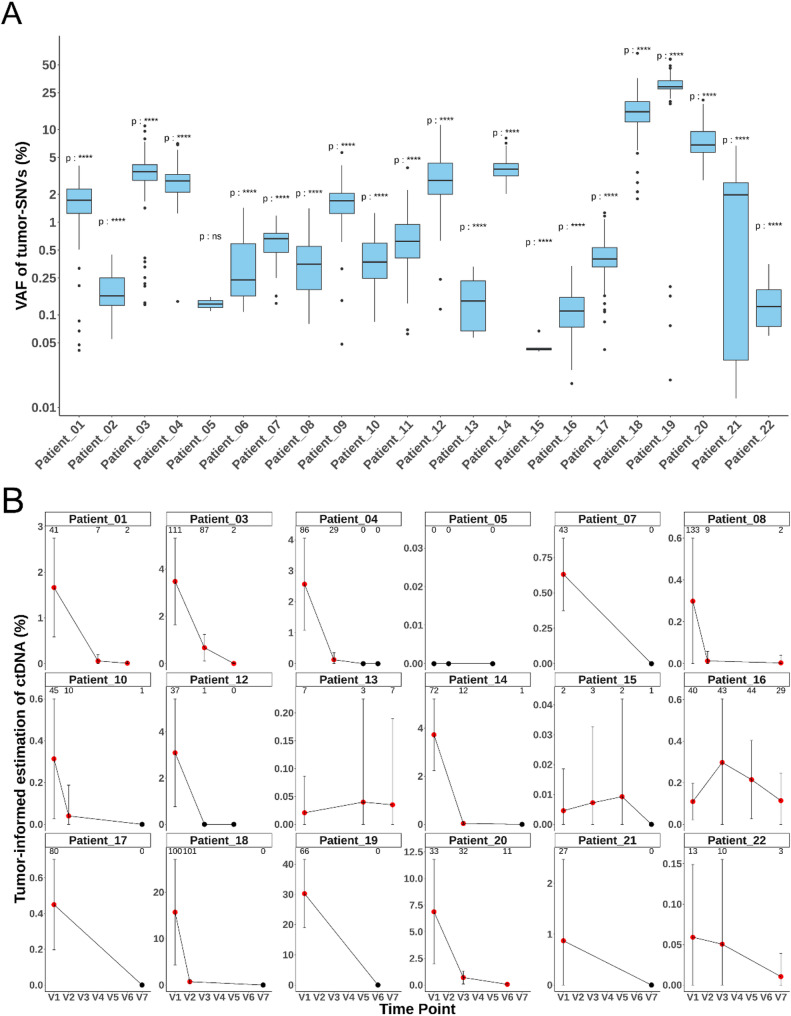
**A**. Distribution of tumor-specific SNV variant allele frequencies (VAFs) in baseline plasma using the tumor-informed approach. Observed VAFs were compared to sequencing error rates, which were estimated for each nucleotide based on the frequency of non-specific SNVs (i.e., genomic positions confirmed to be free of variants in tumor and PBMC samples). The probability of each variant arising from sequencing noise was calculated, and variants with a probability < 1^-^^3^ were considered reliable. ctDNA positivity in sample was defined as the detection of at least two such tumor-specific SNVs. Significance annotations: ns = not significant, **** = *p* < 10 ^6^. **B.** Longitudinal monitoring of ctDNA based on VAFs of tumor-specific SNVs using the tumor-informed method. Only patients with multiple plasma samples available are shown. Error bars indicate the standard deviation of tumor-specific SNVs VAFs. Red points indicate statistically significant ctDNA detection (*p* < 10^-^^6^) compared to background the sequencing noise; black points indicate non-significant values. The number of reliable tumor-specific SNVs detected in each sample is shown at the top of each panel

Figure 2A shows the distribution of tumor-specific SNVs VAF in baseline plasma samples. Of the 22 patients, 21 were ctDNA-positive at baseline, with a mean tumor-SNV VAF of 3.46% (range: 0.01–30.3%). Among the 35 longitudinal plasma samples collected during or at the end of treatment from 18 patients, 21 were ctDNA-positive. Notably, 15 of the 18 patients showed a substantial decrease in ctDNA levels during chemotherapy (Fig. 2B). Six out of 15 patients remained ctDNA-positive at the end of treatment time points (V6–V7).

### Tumor-type informed approach for ctDNA characterization

The tumor-type informed approach was based on training and validating a support vector machine (SVM) classifier. For model development, we defined a training cohort comprising 35 baseline plasmas samples from EOC patients and 24 plasma samples from healthy subjects.

**Fig. 3:**
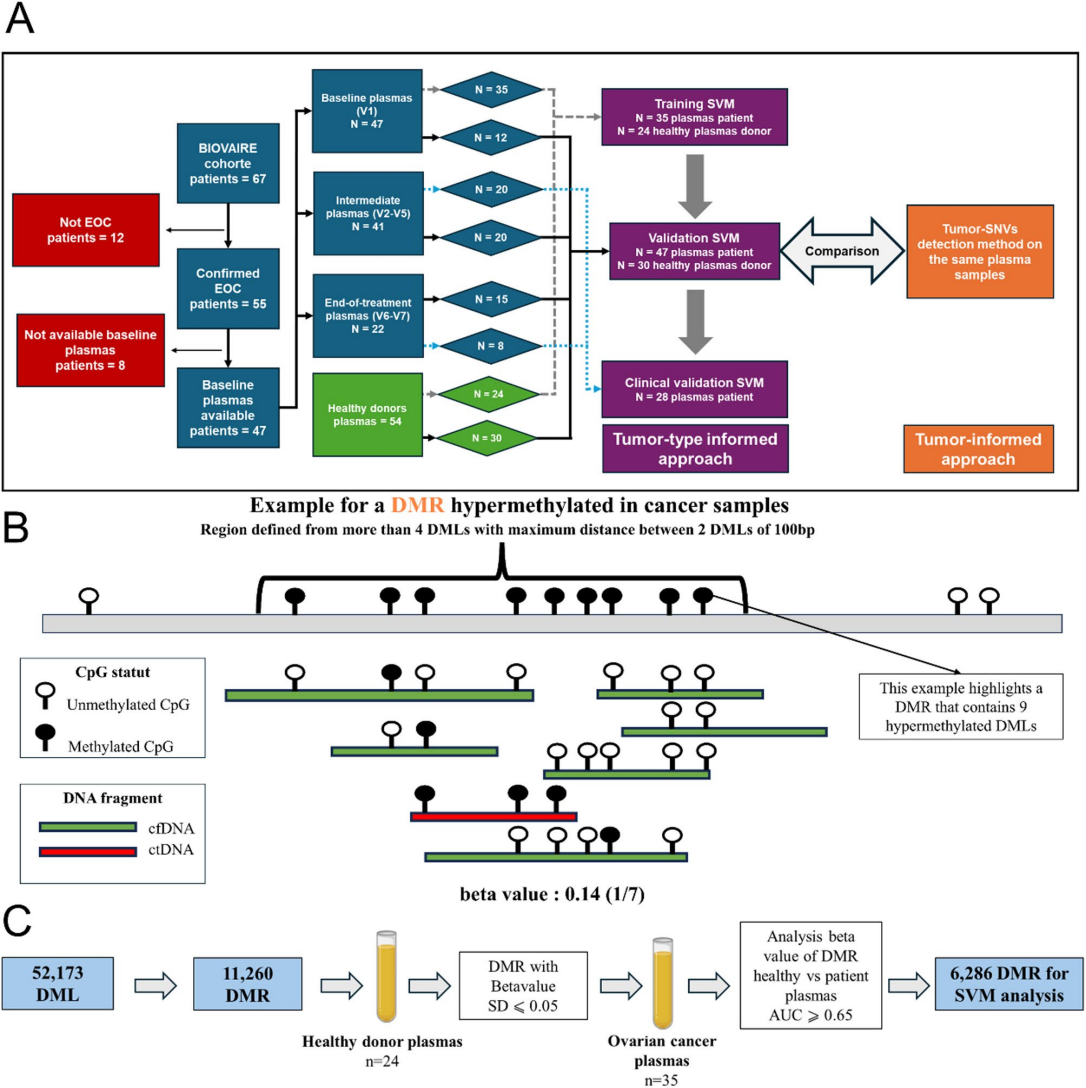
**(A)** Plasma samples workflow. Plasma samples were assigned to different groups for SVM training, validation against the tumor-informed approach, and clinical validation. **(B)** Schematic representation of the fragment-level methylation analysis workflow. Example of a differentially methylated region (DMR) identified as hypermethylated in ovarian tumor tissues compared to healthy ovarian tissues and PBMCs. CfDNA fragments are aligned by genomic coordinates and grouped by their overlap with the DMR. Horizontal bars (red and green) represent individual cfDNA fragments, visualized and classified using wgbstools [17]. The methylation pattern of each fragment across the DMR is analyzed. Fragments with > 85% methylated are classified as tumor-derived (red bar) whereas those with < 85% methylation are considered non-tumor-derived (green bars). The beta value is defined as the proportion of tumor-derived fragments among all cfDNA fragments overlapping the DMR. **(C)** Strategy for DMRs refinement and machine learning analysis. Initially, 11,260 DMRs were identified by comparing ovarian tumor tissues to healthy ovarian tissues and PBMC. These DMRs were first filtered using healthy plasma samples by removing those with a beta values standard deviation > 0.05. The remaining DMRs were then evaluated based on their ability to discriminate between healthy and ovarian cancer plasma samples, using the area under the curve (AUC) of receiver operating characteristic (ROC) analyses. DMRs with an AUC  ≥ 0.65 were retained for SVM analysis

This set was used both for the refinement of DMRs and for training the SVM model. A separate validation cohort was assembled, consisting of 77 plasma samples: 47 from the 22 same EOC patients analyzed using the tumor-informed approach (excluding 10 baseline plasma samples already used in training) and 30 new plasma samples from different healthy individuals. This validation set was used to evaluate the sensitivity and specificity of the SVM classifier and to enable a direct comparison between tumor informed and tumor-type informed strategies (Fig. 3A).

DMRs were defined using DMLs from the tumor-type informed custom panel, based on the principle of local methylation coherence among neighboring CpGs [32]. Briefly, genomic regions were extended 100 bp upstream and downstream of each DML, and regions located within 50 bp of each other were merged. A DMR was retained only if it included at least four CpGs. This approach resulted in the identification of 11,260 DMRs spanning a total of 3.4 Mb.

Following targeted-methylation enrichment and sequencing of cfDNA from plasma samples, we conducted fragment-level methylation analysis (Fig. 3B). Rather than averaging methylation across all fragments at each DML, we focused on the methylation profiles of individual fragments. A cfDNA fragment was classified as tumor-derived if at least 85% of the DMLs it contained matched the methylation pattern observed in EOC tumor samples. To estimate the proportion of tumor-derived fragments in plasma, we computed beta values for each DMR, representing the fraction of fragments exhibiting the tumor-associated methylation pattern. As a first step, we calculated the standard deviation of beta values in plasma samples from the 24 healthy individuals in the training group. DMRs with high variability (standard deviation > 0.05; *n* = 1,528) were excluded to minimize noise. Next, we assessed the discriminative power of each DMR by computing the area under the curve (AUC) from receiver operating characteristic (ROC) analyses comparing healthy and EOC baseline plasma samples.

A total of 6,286 DMRs with AUC ≥ 0.65 were retained, including 1,470 hypermethylated and 4,816 hypomethylated DMRs (Fig. 3C). Across these selected DMRs, baseline EOC plasma samples exhibited elevated beta value relative to healthy controls (Supplemental Fig. 4). These refined DMRs were used to train a SVM model to classify plasma samples as ctDNA-positive or -negative. In this model, baseline EOC plasma samples were used as a surrogate for ctDNA-positive samples, while plasma samples from healthy individuals served as a surrogate for ctDNA-negative samples. For quantitative estimation of ctDNA levels, we defined background noise thresholds using healthy training samples and selected a subset of 201 DMRs. Validation using synthetic samples (*n* = 12) with known tumor DNA quantities demonstrated a strong correlation between expected and measured tumor DNA fractions (*R* = 0.85, *p* < 10^-^^3^; Supplemental Fig. 5).

**Fig. 4:**
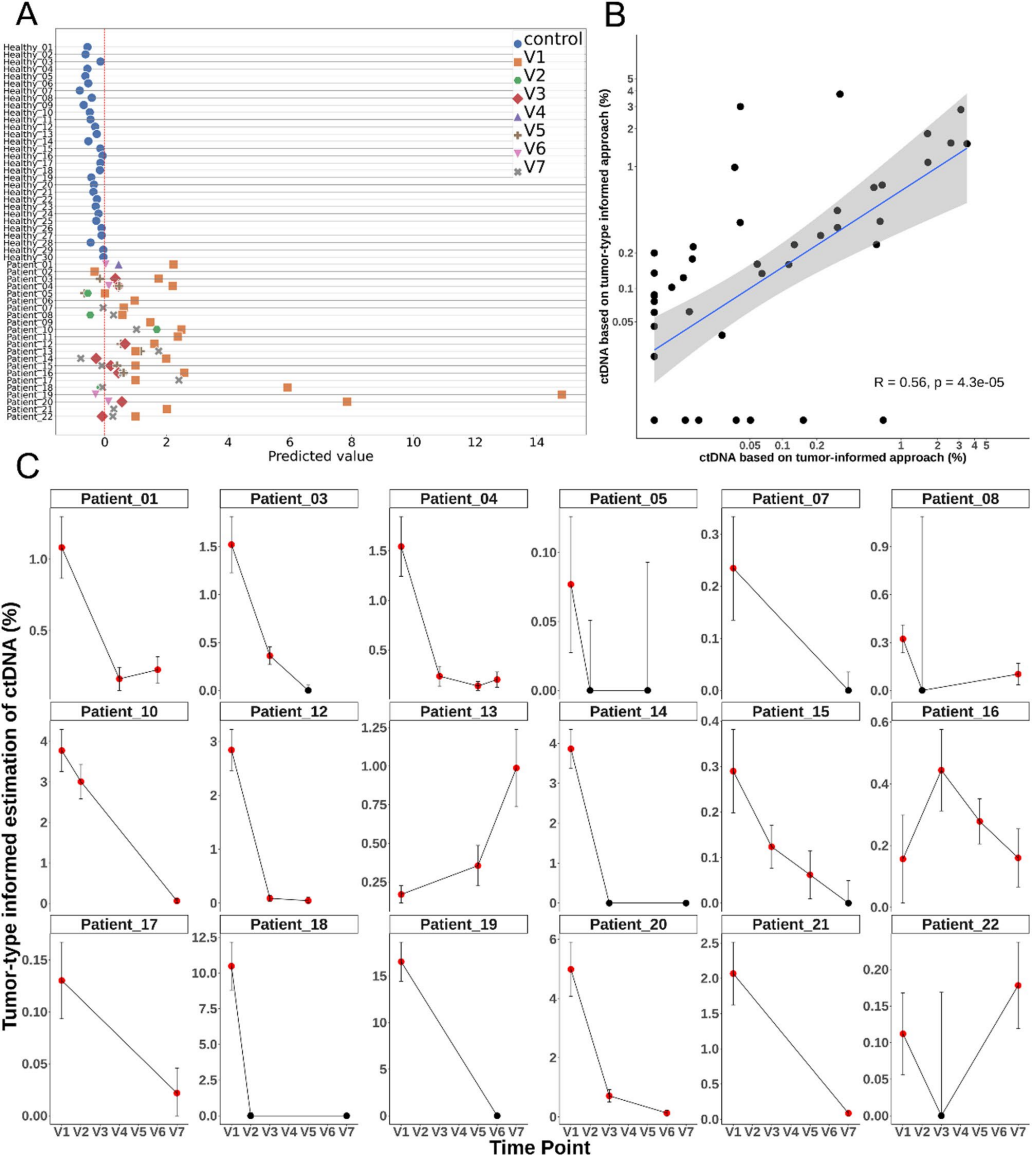
**(A)** Classification of plasma samples using the tumor-type informed SVM model. SVM-predicted values for ctDNA presence are shown for plasma samples previously analyzed with the tumor-informed approach and for 30 healthy donor samples. Each point corresponds to a sample, and the SVM model assigns a score based on the beta values of selected DMRs. The red vertical line indicates the decision boundary (threshold = 0); samples with predicted values above this threshold are classified as ctDNA-positive. Healthy controls are depicted as blue circles. Patient plasma samples are color-coded and shaped according to collection time points (V1 to V7). **(B)** Correlation between ctDNA quantification obtained by the tumor-type informed and tumor-informed approaches. **(C)** Longitudinal monitoring of ctDNA levels using the tumor-type informed approach in patients with multiple plasma samples, previously assessed with the tumor-informed approach. Red points indicate samples classified as ctDNA-positive by the SVM model; black points indicate ctDNA-negative predictions. Error bars represent the 95% confidence intervals (CI95) of beta values derived from the selected DMRs used for tumor-derived DNA quantification

We then applied the SVM model to the validation cohort. All 30 plasma samples from healthy subjects were correctly classified as ctDNA-negative, and 11 out of 12 EOC baseline plasmas were classified as ctDNA-positive, resulting in a theorical sensitivity of 91.7%, specificity of 100% and accuracy of 97.6% (Fig. 4A). The mean ctDNA fraction among positive baseline samples was 1.27% (range: 0.04–3.77%). Notably, the patient negative by the tumor-informed approach was detected as positive by the tumor-type informed method (ctDNA fraction of 0.08%), while one discordant sample positive by the tumor-informed approach (VAF = 0.15%) was negative by the tumor-type informed method. The correlation between ctDNA fractions from both approaches on a set of 47 samples showed a moderate but significant correlation (Spearman’s *R* = 0.56, p = *4.3* × *10*^*−5*^; Fig. 4B). When applying the tumor-type informed approach to follow-up plasma samples (V2–V5; *n* = 20), 13 were classified as ctDNA-positive. At the end of treatment (V6–V7), 10 out of 15 patients remained ctDNA-positive. In 14 patients, ctDNA levels decreased during chemotherapy, consistent with the dynamics observed using the tumor-informed approach. However, among the four patients with stable ctDNA levels according to the tumor-informed approach, the

tumor-type informed method revealed nuanced dynamics: two remained stable, one increase, and one decrease in ctDNA levels (Fig. 4C). Overall, 70.2% of plasma samples (*n* = 33) showed concordant ctDNA classification between the two strategies, while 17% (*n* = 8) were positive only by the tumor-type informed approach, and 13% (*n* = 6) only by the tumor-informed approach (Table 2). These findings support the comparable sensitivity of both approaches (McNemar test, *p* = 0.79) and demonstrated that fragment-level methylation analysis using tumor-type informed DMRs enables ctDNA detection without prior analysis of the patient’s tumor, while requiring fewer sequencing data.

**Table 2:** Contingency table comparing results of tumor-informed and tumor-type informed approaches for ctDNA detection. In the tumor-informed approach, samples are classified as ctDNA-positive when at least 2 tumor-SNVs with a p-value < 10^− 3^ are detected. For the tumor-type informed approach, samples are classified as ctDNA-positive if the predicted value from the SVM classifier is positive

	Positive samples tumor-informed	Negative samples tumor-informed	Total
Positive samples tumor-type informed	26	8	34
Negative samples tumor-type informed	6	7	13
Total	32	15	47

### Over-fitting assessment of the support vector machine classifier

We assessed our machine learning model for potential overfitting using 47 follow-up plasma samples from 25 patients whose baseline plasma samples had been included in the SVM model training set. Among these, 70.2% samples (*n* = 33) were classified as ctDNA-positive, a proportion comparable to the 68.8% (11 out of 16) observed in the follow-up samples from patients not in the training cohort. To evaluate potential biases, we analyzed all validation samples not used for training and assessed whether the input quantity of cfDNA during library preparation influenced the predicted values. No significant correlation was found (Spearman’s *R* = -0.059, *p* = 0.95). However, we observed a tendency for improved discrimination between healthy plasma and patient samples increasing cfDNA input (Supplemental Fig. 6). Finally, we analyzed the potential influence of age on the model by testing for association between beta values and age across the 54 healthy control samples. No significant correlation was detected at the level of individual DMRs (Supplemental Fig. 7).

### Comparative clinical validation of tumor-informed and tumor-type informed approaches for MRD detection

We assessed the ability of the SVM model to detect MRD using the 28 supplementals follow-up plasmas samples from 15 patients (Fig. 3A). These samples had not been analyzed with the tumor-informed approach. Results of ctDNA detection and quantification are presented in Supplemental Fig. 8. Most patients (13 out of the 15) showed a decrease in ctDNA fraction during treatment.

**Fig. 5:**
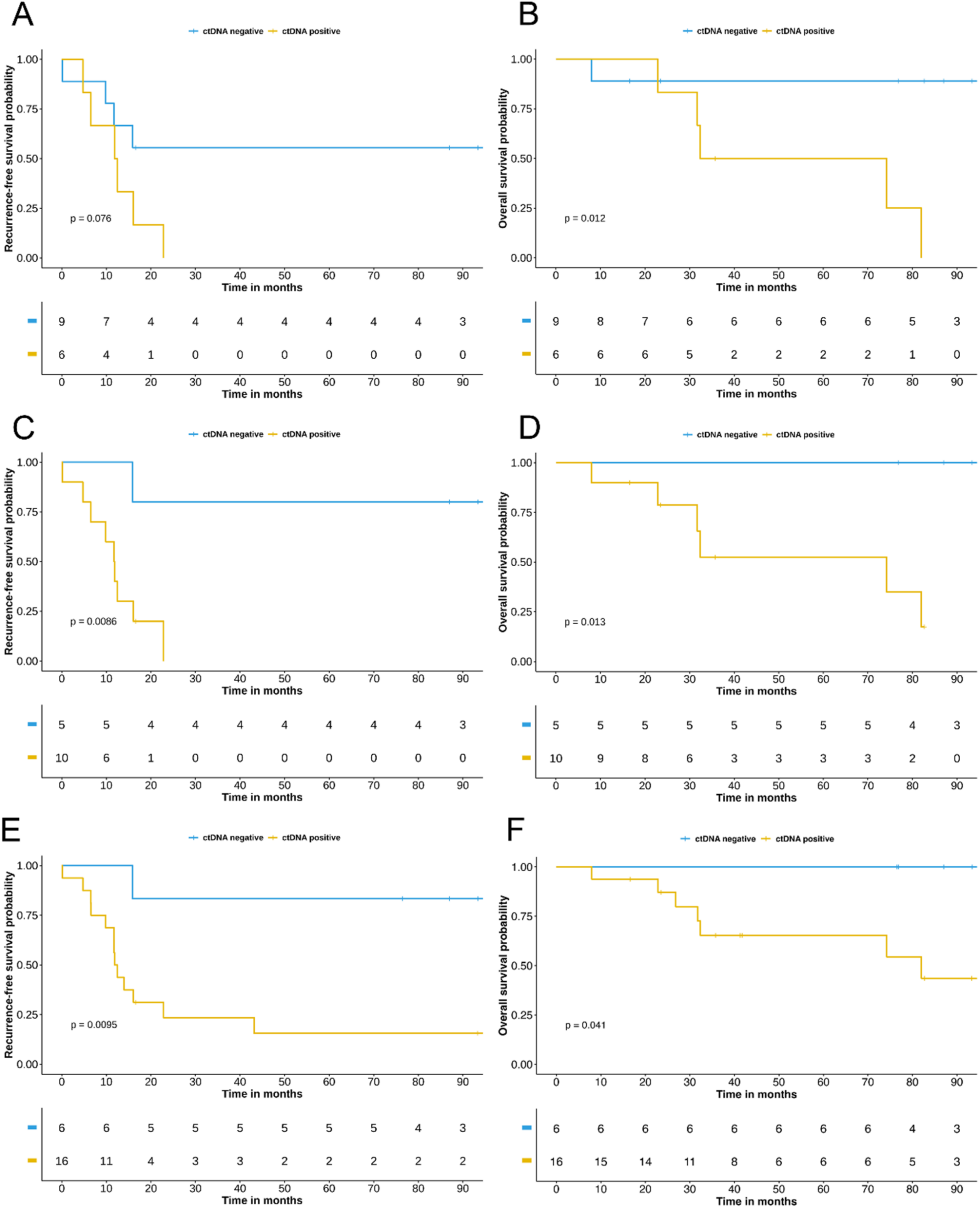
**A**. Progression-Free survival (PFS) based on MRD status defined by the tumor-informed approach. **B**. Overall survival (OS) based on MRD status defined by the tumor-informed approach. **C**. PFS based on MRD status defined by the tumor-type informed approach using the same samples analyzed with the tumor-informed approach. **D**. OS regarding MRD status defined by the tumor-type informed approach using the same samples analyzed with the tumor-informed approach. **E**. PFS based on MRD status defined by the tumor-type informed approach for an extended cohort of 22 patients. **F**. OS based on MRD status defined by the tumor-type informed approach for the same 22 patients

To evaluate the clinical relevance of the tumor-informed and tumor-type informed approaches in predicting outcomes in EOC, we analyzed end-of-treatment plasma samples. Among the 15 patients assessed with both approaches at V6 (*n* = 4) or V7 (*n* = 11), six (40%) were classified as ctDNA-positive by the tumor-informed method, compared to ten (67%) with the tumor-type informed approach. The mean ctDNA fractions were 0.04% and 0.21%, respectively. While the tumor-informed approach did not significantly predict EOC relapse (log-rank *p* = 0.076; Fig. 5A), it was significantly associated with overall survival (OS; log-rank *p* = 0.012; Fig. 5B). In contrast, the tumor-type informed approach significantly predicted both progression-free survival (PFS; log-rank *p* = 0.009; Fig. 5C) and OS (log-rank *p* = 0.013; Fig. 5D).

Expanding the analysis to 22 patients evaluated with the tumor-type informed approach, 16 were classified as ctDNA-positive, of whom 13 experienced relapse with a median PFS of 11 months (95% CI, 6.79–19.60). Among the 6 ctDNA-negative patients, only

one relapsed within 15 months. Kaplan-Meier analysis revealed a significant difference in PFS between ctDNA-positive and ctDNA-negative patients at the end of treatment (log-rank *p* = 0.009; hazard ratio = 9.44; 95% CI 1.22–73.26; Fig. 5E). Furthermore, none of the ctDNA-negative died during follow-up, while 6 out of the 16 of ctDNA-positive patients (37.5%) succumbed during the surveillance period. Kaplan-Meier analysis also revealed a significant difference in OS between ctDNA-positive and ctDNA-negative groups (log-rank *p* = 0.041; Fig. 5F). Overall, our tumor-type informed SVM model effectively predicted both relapses and overall survival, while requiring fewer sequencing data than the tumor-informed approach.

To benchmark the model against conventional clinical response criteria, we evaluated both CA125 normalization and cytoreduction score. Among patients with available data, neither CA125 normalization (*n* = 15; Fisher’s exact test *p* = 0.52; Supplementary Table 1) nor cytoreduction score (*n* = 19; Fisher’s exact test *p* = 0.33; Supplementary Table 2) significantly predicted relapse. Finally, we compared ctDNA-based prediction of relapse with computed tomography (CT) assessment of residual disease at the end of treatment. Among the 19 patients with available CT-scan data after the completion of first line treatment, detection of residual cancer lesions showed a significant association with EOC relapse (Fisher’s exact test *p* = 0.044; Supplementary Table 3). Six out of the 12 who relapse showed evidence of persistent cancerous lesions on CT-scan. When comparing lesion detection by CT-scan with the tumor-type informed ctDNA detection approach, the latter demonstrated improved sensitivity in predicting EOC relapse (Supplementary Table 4; McNemar’s test *p* = 0.04). These findings suggest that ctDNA detection at the end of treatment using the tumor-type informed approach outperforms radiological and conventional biological markers in forecasting EOC recurrence.

## Discussion

Highly sensitive methods are required for the detection and quantification of ctDNA in the context of MRD assessment. In this study, we compared two strategies: a tumor-informed approach, based on somatic SNVs identified from each patient’s tumor, and a tumor-type informed approach, which leverage DMRs common in EOC samples. Both approaches were applied to a cohort of 47 patients from the Biovaire study, benefiting from extended clinical follow-up, with some patients monitored for up to 100 months after completion of first-line therapy.

In the tumor-informed approach, WES was performed on tumor samples, using matched germline DNA from PBMCs as a reference to identify tumor-specific SNVs. The median number of SNVs identified in our cohort (median: 66; IQR: 50–83) was consistent with observations from TCGA EOC samples (median: 62.8; IQR: 34–77) [33–38]. Most tumors harbored mutations in commonly altered EOC genes, such as *TP53*, *TTN*, and *CSMD3* [39, 40]. However, this relatively limited number of SNVs presents a challenge for ctDNA detection, especially when plasma input in low. In 22 baseline plasma samples analyzed using the tumor-informed approach, we extracted an average of 14.5 ng cfDNA, representing approximately 4,500 genome equivalents (GE). According to Zviran *et al*.., under these conditions the probability of detecting ctDNA at a 10^-^^4^ fraction ranges from 80 to 100%, which aligns with our detection rate of 21 out of 22 samples [16]. The single negative sample had one of the lowest cfDNA input (6.1 ng) for library preparation. In follow-up plasma samples, with an average cfDNA input of 5.6 ng, ctDNA was detected in 21 out of 35 samples. Sensitivity could potentially be improved by increasing cfDNA input (requiring larger blood volumes) or targeting variant types more robust to sequencing error, such as indels or phased variants. However, this would substantially increase sequencing costs, particularly due to the need for WGS of the tumor to identify such variants [41, 42]. These challenges underscore the importance of optimizing the number and nature of markers used for ctDNA tracking.

To address this, we evaluated whether a tumor-type informed approach based on cfDNA methylation could enhance detection sensitivity while remaining cost-effective. Existing public methylation datasets, mostly derived from the Illumina 27 K array, offer limited utility for biomarkers discovery. We therefore generated a comprehensive dataset using a targeted methylation panel covering approximately 14% of the CpGs in the human genome. To improve robustness, we also incorporated two WGBS datasets from healthy ovarian tissues available through ENCODE. From these data, we identified 6,286 DMRs and designed a custom sequencing panel. Several genes associated with these regions, such as *CELF2*,* FAIM2*,* SIM2*,* CDO1*, and *C2CD4D*, have already been described as potential EOC biomarkers, while others, such as *JAK3* and *SOX2*, are known oncogenic drivers [43–45]. With over 6,000 targeted markers, the theoretical limit of detection using this panel extends to 10^-^^5^ for 2,000 GE of cfDNA input.

When comparing both approaches on baseline plasma samples, ctDNA was detected in 95.4% of samples using the tumor-informed method and 97.9% (including training samples) using the tumor-type informed approach. On follow-up samples, the tumor-type-informed approach remained comparable in sensitivity, detecting ctDNA in 23 out of 35 cases versus 21 by the tumor-informed method. This innovative approach provides several advantages: it eliminates the need for tumor sequencing, significantly reduces turnaround time, and cuts sequencing costs by a factor of eight while retaining sensitivity to detect low ctDNA fractions.

In addition, fragment-level methylation analysis strengthened ctDNA detection by reducing reliance on unmethylated cytosine conversion rates and sequencing errors [28]. By aggregating methylation information across multiple CpGs per cfDNA fragment and integrating an SVM classifier, we achieved 100% specificity in ctDNA detection. Furthermore, in a clinical setting, the tumor-type informed approach achieved a sensitivity of 92.3% for MRD detection at the end of treatment: among 13 patients who relapsed, 12 were classified as ctDNA-positive. The median time to relapse in this group was 11 months. Although specificity was lower (55.6%), it is important to note that two ctDNA-positive patients were lost to follow-up before reaching two years.

This study has some limitations. The DMRs used to design our methylation panel were derived from a relatively small discovery cohort of 12 EOC tissues, 12 matched PBMC samples, and 9 healthy ovarian tissues. Although we studied a limited number of patient tumors to identify our DMRs, which does not fully protect against inter-individual variability in tumor profiles, the overall sensitivity of our method for detecting ctDNA at baseline is 97.9%, supporting the robustness of the selected markers. Although we controlled for age, cfDNA input, and patient variability, overfitting of the SVM model cannot be entirely ruled out, although we believe this risk to be low. Finally, MRD prediction at the end of treatment was evaluated in a cohort of only 22 patients, and larger independent studies will be necessary to validate these findings before advancing to clinical trials.

Compared to other recent efforts, our tumor-type informed approach demonstrated similar or superior performance. Hou *et al.* achieved comparable sensitivity and specificity using deep WES and personalized amplicon-based monitoring [46]. In contrast, the tumor-naïve assay developed by Hoe *et al.*, which monitors ctDNA through nine frequently mutated genes, detected ctDNA in only 69.2% of baseline samples. While their method accurately predicted relapse in many cases, some patients classified as negative ultimately relapsed, underscoring a notable lack of sensitivity [13].

CA125 is commonly used to monitor peritoneal involvement in EOC, but it provides an imprecise assessment of tumor burden and is not specific of EOC lesions [6]. The decrease in CA125 during neoadjuvant chemotherapy, particularly when measured using the KELIM score, serves as a marker of chemosensitivity. However, CA125 lacks specificity and is not suitable for measuring residual disease at the end of first-line chemotherapy [47]. In clinical practice, a ctDNA assay could be routinely performed on a plasma sample at diagnosis, during first-line chemotherapy and at the end of treatment (after six cycles of chemotherapy and cytoreductive surgery) to assess residual disease. Our preliminary results suggest that the presence of ctDNA could be effective for this specific indication. Additionally, ctDNA measurement could be used during surveillance to detect recurrence. However, the clinical impact of ctDNA-based assessment—both for minimal residual disease and for surveillance—requires further evaluation. The tumor-type informed approach is cost-effective, time-efficient, and eliminates the need for tumor biopsies. It is also adaptable to other cancer types and could be a valuable tool for evaluating treatment responses in clinical assays.

## Conclusion

By analyzing cfDNA methylation patterns in plasma from healthy donors and EOC patients, we achieved improved sensitivity for detecting MRD and predicting relapse compared to a traditional tumor-informed approach. Notably, this approach allows classification of plasma samples regarding ctDNA presence without requiring prior knowledge of the patient’s tumor SNVs. It achieves this purpose with eightfold less data than tumor-informed strategies that rely on WES of the patient’s tumor and mutation tracking in plasma. This cost-effective and non-invasive method holds significant potential to improve EOC patient care. For patients deemed free of MRD following first-line treatment, a de-escalation of maintenance therapy can be envisioned. Conversely, MRD-positive patients could benefit from closer monitoring and tailored interventions, potentially improving outcomes. Future studies should validate these findings in larger, multi-center cohorts, paving the way for broader clinical implementation.

## Electronic supplementary material

Below is the link to the electronic supplementary material.

Supplementary Material 1

## Data Availability

The datasets used and/or analyzed during the current study are available from the corresponding author on reasonable request.

## References

[1] Sung H, Ferlay J, Siegel RL, Laversanne M, Soerjomataram I, Jemal A, et al. Global Cancer statistics 2020: GLOBOCAN estimates of incidence and mortality worldwide for 36 cancers in 185 countries. CA Cancer J Clin. 2021;71(3):209–49. 33538338 10.3322/caac.21660

[2] Hennessy BT, Coleman RL, Markman M. Ovarian cancer. Lancet. 2009;374(9698):1371–82. 19793610 10.1016/S0140-6736(09)61338-6

[3] Berek JS, Renz M, Kehoe S, Kumar L, Friedlander M. Cancer of the ovary, fallopian tube, and peritoneum: 2021 update. Int J Gynaecol Obstet. 2021;155(1):61–85. 34669199 10.1002/ijgo.13878 PMC9298325

[4] du Bois A, Reuss A, Pujade-Lauraine E, Harter P, Ray-Coquard I, Pfisterer J. Role of surgical outcome as prognostic factor in advanced epithelial ovarian cancer: a combined exploratory analysis of 3 prospectively randomized phase 3 multicenter trials: by the arbeitsgemeinschaft gynaekologische onkologie studiengruppe ovarialkarzinom (AGO-OVAR) and the groupe d’investigateurs Nationaux pour les etudes des cancers de l’ovaire (GINECO). Cancer. 2009;115(6):1234–44. 19189349 10.1002/cncr.24149

[5] Torre LA, Trabert B, DeSantis CE, Miller KD, Samimi G, Runowicz CD, et al. Ovarian cancer statistics, 2018. CA Cancer J Clin. 2018;68(4):284–96. 29809280 10.3322/caac.21456 PMC6621554

[6] Zhang M, Cheng S, Jin Y, Zhao Y, Wang Y. Roles of CA125 in diagnosis, prediction, and oncogenesis of ovarian cancer. Biochim Biophys Acta Rev Cancer. 2021;1875(2):188503. 33421585 10.1016/j.bbcan.2021.188503

[7] Pantel K, Alix-Panabières C. Liquid biopsy and minimal residual disease - latest advances and implications for cure. Nat Rev Clin Oncol. 2019;16(7):409–24. 30796368 10.1038/s41571-019-0187-3

[8] Lo YMD, Han DSC, Jiang P, Chiu RWK. Epigenetics, fragmentomics, and topology of cell-free DNA in liquid biopsies. Science. 2021;372(6538):eaaw3616. 33833097 10.1126/science.aaw3616

[9] Azaïs H, Brochard C, Taly V, Benoit L, Ferron G, Ray-Coquard I, et al. Prognostic value of Circulating tumor DNA at diagnosis and its early decrease after one cycle of neoadjuvant chemotherapy for patients with advanced epithelial ovarian cancer. An ancillary analysis of the CHIVA phase II GINECO trial. Gynecol Oncol. 2025;192:145–54. 39671779 10.1016/j.ygyno.2024.12.004

[10] Laude É, Azaïs H, Ben Sassi M, Bats AS, Taly V, Laurent-Puig P. Clinical value of Circulating tumor DNA for patients with epithelial ovarian cancer. Int J Gynecol Cancer. 2025;35(7):101925. 40424838 10.1016/j.ijgc.2025.101925

[11] Kossaï M, Leary A, Scoazec JY, Genestie C. Ovarian cancer: A heterogeneous disease. Pathobiology. 2018;85(1–2):41–9. 29020678 10.1159/000479006

[12] Dong Q, Chen C, Hu Y, Zhang W, Yang X, Qi Y, et al. Clinical application of molecular residual disease detection by circulation tumor DNA in solid cancers and a comparison of technologies: review Article. Cancer Biol Ther. 2023;24(1):2274123. 37955635 10.1080/15384047.2023.2274123 PMC10653633

[13] Heo J, Kim YN, Shin S, Lee K, Lee JH, Lee YJ, et al. Serial Circulating tumor DNA analysis with a tumor-Naïve Next-Generation sequencing panel detects minimal residual disease and predicts outcome in ovarian Cancer. Cancer Res. 2024;84(3):468–78. 38038965 10.1158/0008-5472.CAN-23-1429

[14] Razavi P, Li BT, Brown DN, Jung B, Hubbell E, Shen R, et al. High-intensity sequencing reveals the sources of plasma Circulating cell-free DNA variants. Nat Med. 2019;25(12):1928–37. 31768066 10.1038/s41591-019-0652-7 PMC7061455

[15] Stadler JC, Belloum Y, Deitert B, Sementsov M, Heidrich I, Gebhardt C, et al. Current and future clinical applications of ctDNA in Immuno-Oncology. Cancer Res. 2022;82(3):349–58. 34815256 10.1158/0008-5472.CAN-21-1718 PMC9397642

[16] Zviran A, Schulman RC, Shah M, Hill STK, Deochand S, Khamnei CC, et al. Genome-wide cell-free DNA mutational integration enables ultra-sensitive cancer monitoring. Nat Med. 2020;26(7):1114–24. 32483360 10.1038/s41591-020-0915-3 PMC8108131

[17] Chalmers ZR, Connelly CF, Fabrizio D, Gay L, Ali SM, Ennis R, et al. Analysis of 100,000 human cancer genomes reveals the landscape of tumor mutational burden. Genome Med. 2017;9:34. 28420421 10.1186/s13073-017-0424-2 PMC5395719

[18] Dai X, Ren T, Zhang Y, Nan N. Methylation multiplicity and its clinical values in cancer. Expert Rev Mol Med. 2021;23:e2. 33787478 10.1017/erm.2021.4 PMC8086398

[19] Luo H, Wei W, Ye Z, Zheng J, Xu RH. Liquid biopsy of methylation biomarkers in Cell-Free DNA. Trends Mol Med. 2021;27(5):482–500. 33500194 10.1016/j.molmed.2020.12.011

[20] Liang L, Zhang Y, Li C, Liao Y, Wang G, Xu J, et al. Plasma CfDNA methylation markers for the detection and prognosis of ovarian cancer. eBioMedicine. 2022;83:104222. 35973389 10.1016/j.ebiom.2022.104222 PMC9396542

[21] Liu MC, Oxnard GR, Klein EA, Swanton C, Seiden MV, Liu MC, et al. Sensitive and specific multi-cancer detection and localization using methylation signatures in cell-free DNA. Ann Oncol. 2020;31(6):745–59. 33506766 10.1016/j.annonc.2020.02.011 PMC8274402

[22] Garrigou S, Perkins G, Garlan F, Normand C, Didelot A, Le Corre D, et al. A study of hypermethylated Circulating tumor DNA as a universal colorectal Cancer biomarker. Clin Chem. 2016;62(8):1129–39. 27251038 10.1373/clinchem.2015.253609

[23] Beinse G, Borghese B, Métairie M, Just PA, Poulet G, Garinet S, et al. Highly specific Droplet-Digital PCR detection of universally methylated Circulating tumor DNA in endometrial carcinoma. Clin Chem. 2022;68(6):782–93. 35323926 10.1093/clinchem/hvac020

[24] Vaisvila R, Ponnaluri VKC, Sun Z, Langhorst BW, Saleh L, Guan S, et al. Enzymatic Methyl sequencing detects DNA Methylation at single-base resolution from picograms of DNA. Genome Res. 2021;31(7):1280–9. 34140313 10.1101/gr.266551.120 PMC8256858

[25] Feng H, Conneely KN, Wu H. A bayesian hierarchical model to detect differentially methylated loci from single nucleotide resolution sequencing data. Nucleic Acids Res. 2014;42(8):e69. 24561809 10.1093/nar/gku154 PMC4005660

[26] Akalin A, Kormaksson M, Li S, Garrett-Bakelman FE, Figueroa ME, Melnick A, et al. MethylKit: a comprehensive R package for the analysis of genome-wide DNA methylation profiles. Genome Biol. 2012;13(10):R87. 23034086 10.1186/gb-2012-13-10-r87 PMC3491415

[27] Korthauer K, Chakraborty S, Benjamini Y, Irizarry RA. Detection and accurate false discovery rate control of differentially methylated regions from whole genome bisulfite sequencing. Biostatistics. 2019;20(3):367–83. 29481604 10.1093/biostatistics/kxy007 PMC6587918

[28] Li W, Li Q, Kang S, Same M, Zhou Y, Sun C, et al. CancerDetector: ultrasensitive and non-invasive cancer detection at the resolution of individual reads using cell-free DNA methylation sequencing data. Nucleic Acids Res. 2018;46(15):e89. 29897492 10.1093/nar/gky423 PMC6125664

[29] Loyfer N, Rosenski J, Kaplan T. wgbstools: A computational suite for DNA methylation sequencing data representation, visualization, and analysis. 2024. 10.26508/lsa.202503514 PMC12861688 41611450

[30] Tarasov A, Vilella AJ, Cuppen E, Nijman IJ, Prins P. Sambamba: fast processing of NGS alignment formats. Bioinformatics. 2015;31(12):2032–4. 25697820 10.1093/bioinformatics/btv098 PMC4765878

[31] Loyfer N, Magenheim J, Peretz A, Cann G, Bredno J, Klochendler A, et al. A DNA methylation atlas of normal human cell types. Nature. 2023;613(7943):355–64. 36599988 10.1038/s41586-022-05580-6 PMC9811898

[32] Affinito O, Palumbo D, Fierro A, Cuomo M, De Riso G, Monticelli A, et al. Nucleotide distance influences co-methylation between nearby CpG sites. Genomics. 2020;112(1):144–50. 31078719 10.1016/j.ygeno.2019.05.007

[33] Berger AC, Korkut A, Kanchi RS, Hegde AM, Lenoir W, Liu W, et al. A comprehensive Pan-Cancer molecular study of gynecologic and breast cancers. Cancer Cell. 2018;33(4):690–e7059. 29622464 10.1016/j.ccell.2018.03.014 PMC5959730

[34] de Bruijn I, Kundra R, Mastrogiacomo B, Tran TN, Sikina L, Mazor T, et al. Analysis and visualization of longitudinal genomic and clinical data from the AACR project GENIE biopharma collaborative in cBioPortal. Cancer Res. 2023;83(23):3861–7. 37668528 10.1158/0008-5472.CAN-23-0816 PMC10690089

[35] Elkin R, Oh JH, Liu YL, Selenica P, Weigelt B, Reis-Filho JS, et al. Geometric network analysis provides prognostic information in patients with high grade serous carcinoma of the ovary treated with immune checkpoint inhibitors. NPJ Genom Med. 2021;6(1):99. 34819508 10.1038/s41525-021-00259-9 PMC8613272

[36] Cancer Genome Atlas Research Network. Integrated genomic analyses of ovarian carcinoma. Nature. 2011;474(7353):609–15. 21720365 10.1038/nature10166 PMC3163504

[37] Gao J, Aksoy BA, Dogrusoz U, Dresdner G, Gross B, Sumer SO, et al. Integrative analysis of complex cancer genomics and clinical profiles using the cBioPortal. Sci Signal. 2013;6(269):pl1. 23550210 10.1126/scisignal.2004088 PMC4160307

[38] Cerami E, Gao J, Dogrusoz U, Gross BE, Sumer SO, Aksoy BA, et al. The cBio cancer genomics portal: an open platform for exploring multidimensional cancer genomics data. Cancer Discov. 2012;2(5):401–4. 22588877 10.1158/2159-8290.CD-12-0095 PMC3956037

[39] Lu N, Liu J, Xu M, Liang J, Wang Y, Wu Z, et al. CSMD3 is associated with tumor mutation burden and immune infiltration in ovarian Cancer patients. Int J Gen Med. 2021;14:7647–57. 34764678 10.2147/IJGM.S335592 PMC8575319

[40] Ma MC, Lavi ES, Altwerger G, Lin ZP, Ratner ES. Predictive modeling of gene mutations for the survival outcomes of epithelial ovarian cancer patients. PLoS ONE. 2024;19(7):e0305273. 38976671 10.1371/journal.pone.0305273 PMC11230535

[41] Fernandez SV, Tan YF, Rao S, Fittipaldi P, Sheriff F, Borghaei H, et al. Validation of a molecular diagnostic test for Circulating tumor DNA by Next-Gen sequencing. Int J Mol Sci. 2023;24(21):15779. 37958763 10.3390/ijms242115779 PMC10648112

[42] Kurtz DM, Soo J, Co Ting Keh L, Alig S, Chabon JJ, Sworder BJ, et al. Enhanced detection of minimal residual disease by targeted sequencing of phased variants in Circulating tumor DNA. Nat Biotechnol. 2021;39(12):1537–47. 34294911 10.1038/s41587-021-00981-w PMC8678141

[43] Marinelli LM, Kisiel JB, Slettedahl SW, Mahoney DW, Lemens MA, Shridhar V, et al. Methylated DNA markers for plasma detection of ovarian cancer: discovery, validation, and clinical feasibility. Gynecol Oncol. 2022;165(3):568–76. 35370009 10.1016/j.ygyno.2022.03.018 PMC9133226

[44] Kallio HM, Savolainen K, Virtanen T, Ryyppö L, Selin H, Martikainen P, et al. Sensitive Circulating tumor DNA-based residual disease detection in epithelial ovarian cancer. Life Sci Alliance. 2024;7(6):e202402658. 38580393 10.26508/lsa.202402658 PMC10997860

[45] Senturk Kirmizitas T, van den Berg C, Boers R, Helmijr J, Makrodimitris S, Dag HH, et al. Epigenetic and genomic hallmarks of PARP-Inhibitor resistance in ovarian Cancer patients. Genes (Basel). 2024;15(6):750. 38927686 10.3390/genes15060750 PMC11203368

[46] Hou JY, Chapman JS, Kalashnikova E, Pierson W, Smith-McCune K, Pineda G, et al. Circulating tumor DNA monitoring for early recurrence detection in epithelial ovarian cancer. Gynecol Oncol. 2022;167(2):334–41. 36117009 10.1016/j.ygyno.2022.09.004

[47] Lauby A, Colomban O, Corbaux P, Peron J, Van Wagensveld L, Gertych W, et al. The increasing prognostic and predictive roles of the tumor primary chemosensitivity assessed by CA-125 elimination rate constant K (KELIM) in ovarian cancer: A narrative review. Cancers (Basel). 2021;14(1):98. 35008262 10.3390/cancers14010098 PMC8750686

