# Supplementary material for: Improved tumor-type informed compared to tumor-informed mutation tracking for ctDNA detection and microscopic residual disease assessment in epithelial ovarian cancer
Source: J Exp Clin Cancer Res. 2025 Jun 12;44:174. doi: 10.1186/s13046-025-03433-4 (PMC12160408; doi:10.1186/s13046-025-03433-4)
Supplement: Supplementary file 1 — Supplementary Material 1 [file 13046_2025_3433_MOESM1_ESM.docx]

**Supplementals**


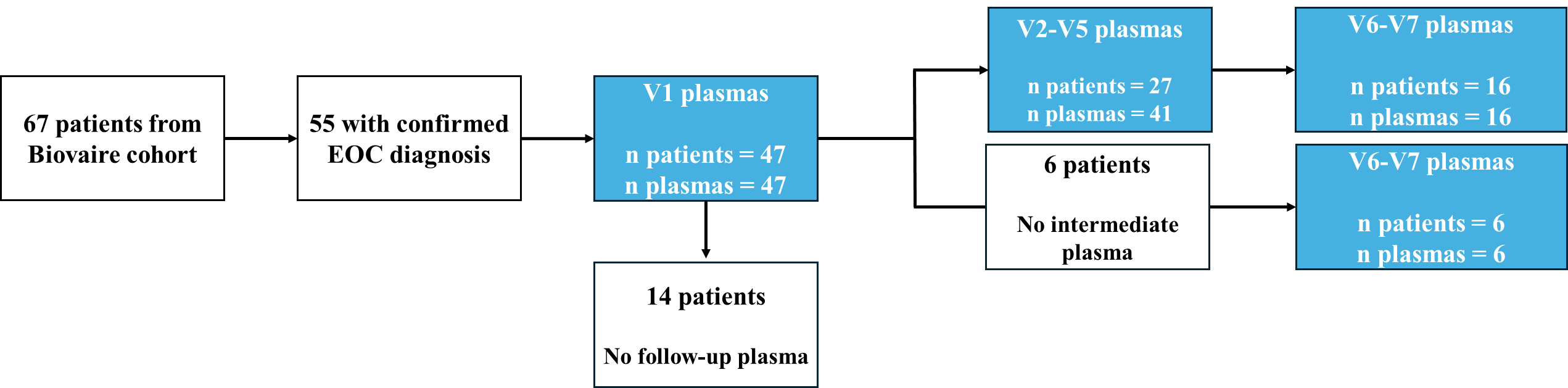


**Supplementary figure 1**: Consort diagram of plasma samples used in the study.


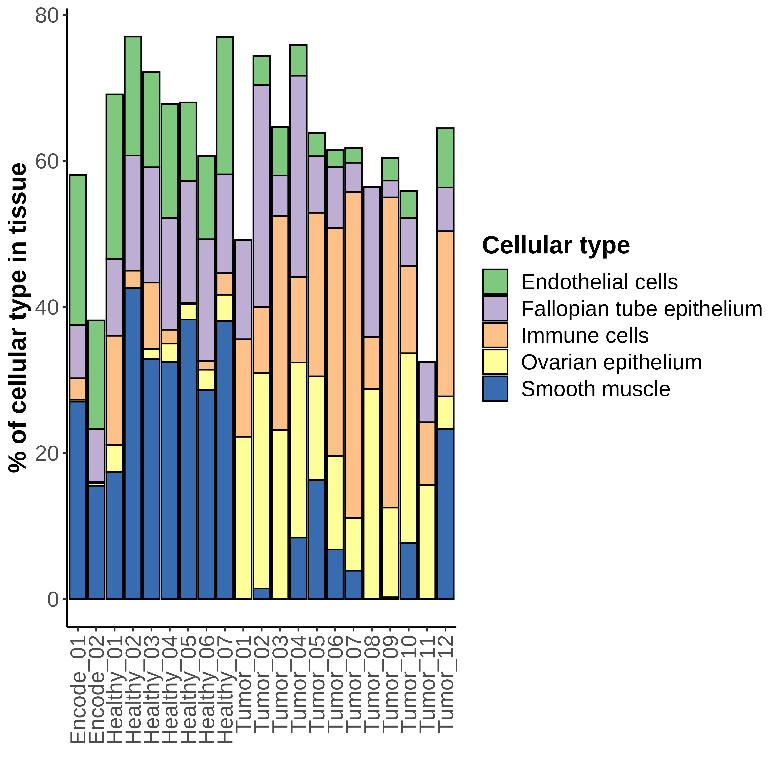


**Supplementary figure 2**: Deconvolution of methylation signal from normal ovarian tissues (n = 9) and ovarian tumors (n = 12) using *UXM_deconv* algorithm (16).


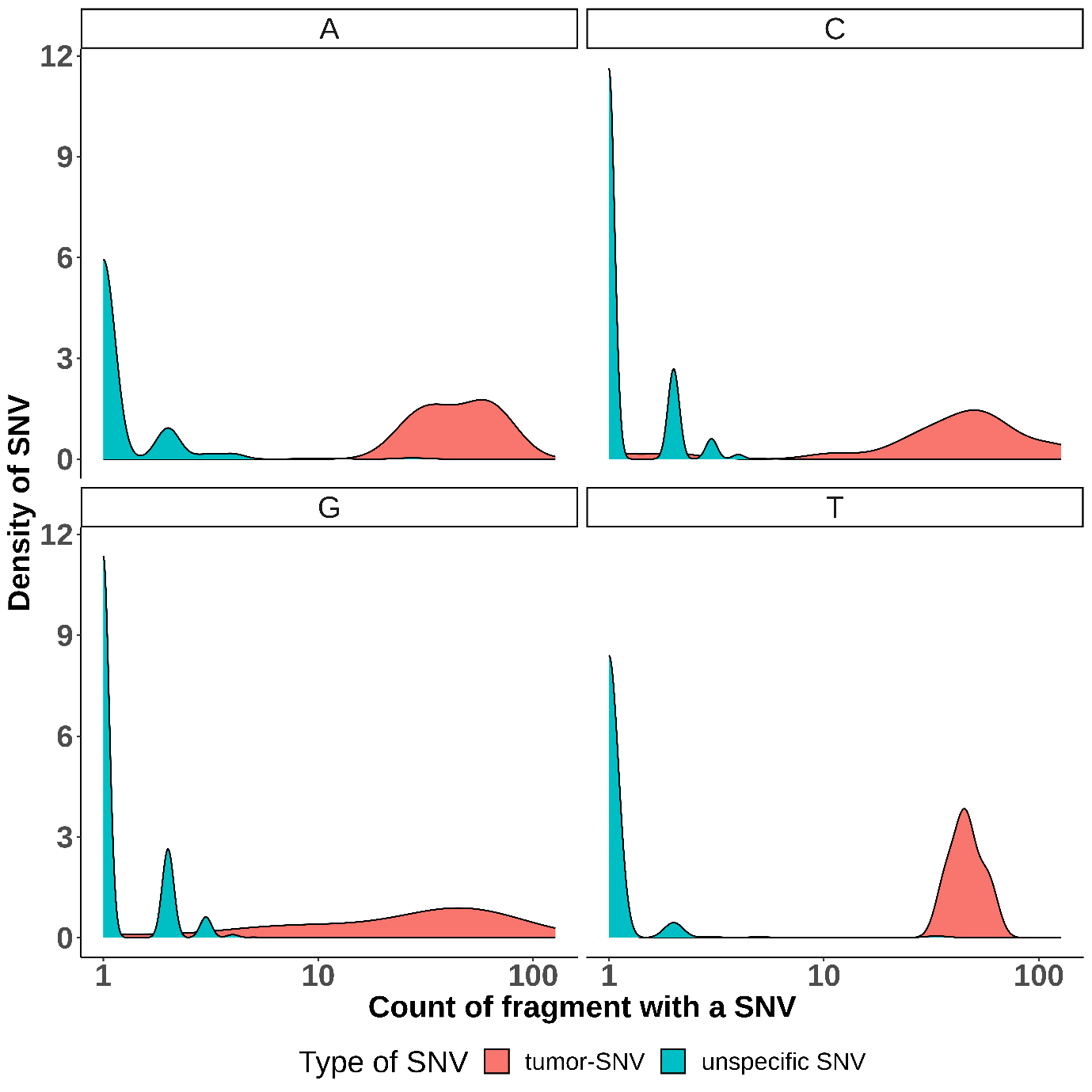


**Supplementary figure 3:** This density plot illustrates the distribution of fragment counts for expected variants (tumor-SNVs, shown in red) versus unspecific variants (shown in blue) for a patient before the beginning of the chemotherapy (V1). Expected SNVs are supported by multiple fragments (up to 127), whereas unspecific SNVs are typically supported by only a few fragments (1 to 4). Additionally, sequencing errors are more frequent at cytosine and guanosine bases. A statistical analysis was conducted to compare the frequencies of unspecific and expected SNVs by base type, facilitating the distinction of true ctDNA signals and potential sequencing artifacts.


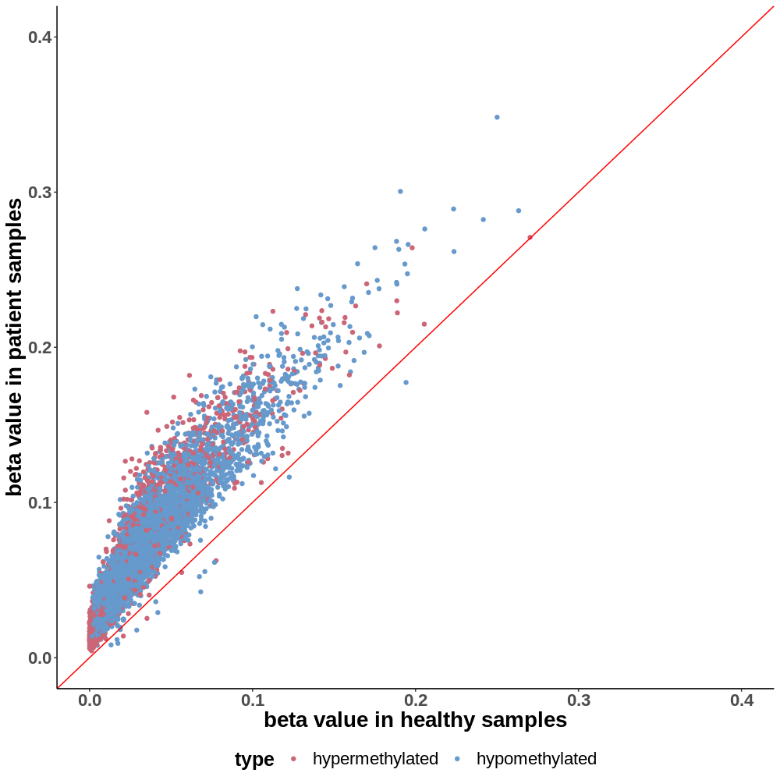


**Supplementary figure 4:** Scatter plot illustrating the correlation of average beta values between healthy and baseline patient plasma samples for each retained DMR. Tumor hypermethylated DMRs are shown in red, while tumor hypomethylated DMRs are shown in blue, highlighting the increasing of beta value in the tumor plasma patients as compared to healthy plasma subject.


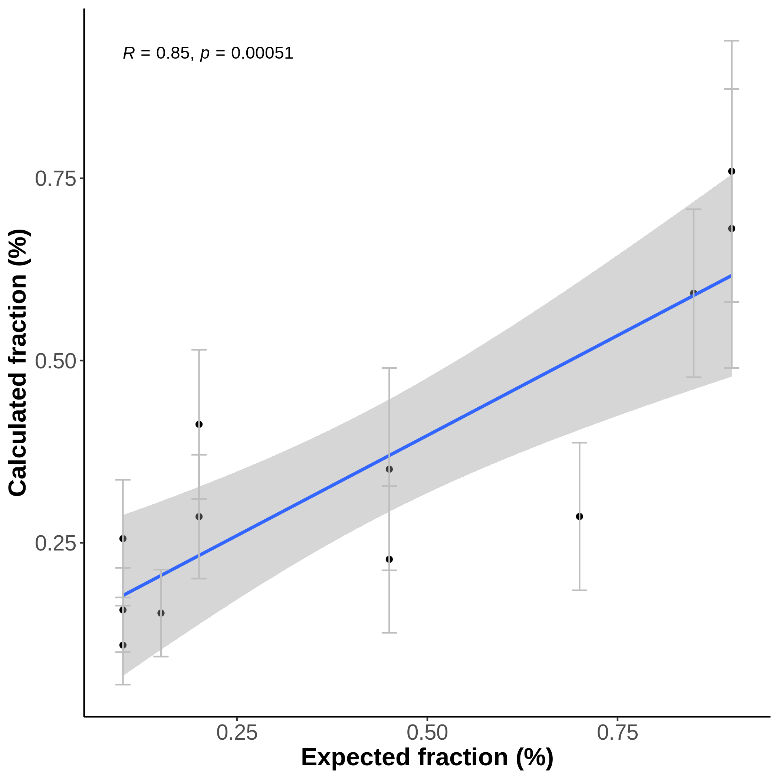


**Supplementary figure 5:** Correlation between tumoral DNA quantified by beta values of selected DMRs and expected values from in-house mixes of DNA (n = 12). Error bars represent the 95% confidence intervals of the beta values from DMRs used for tumoral DNA quantification.


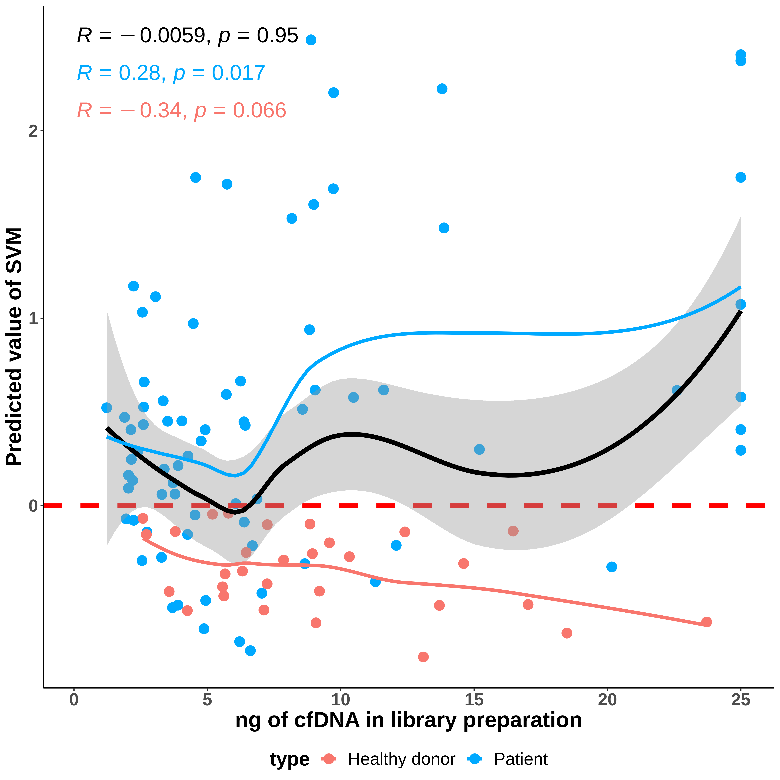


**Supplementary figure 6**: Correlation between cfDNA input in library preparation and SVM model predicted values. EOC patient plasmas are represented in blue (n = 75), while healthy subject plasmas are in red (n = 30). The horizontal red dashed line represents the prediction value threshold for sample classification. The black line shows the correlation between predicted values and input amounts for all samples, while the blue line corresponds to patient plasma samples and the red line to healthy subject plasma samples.


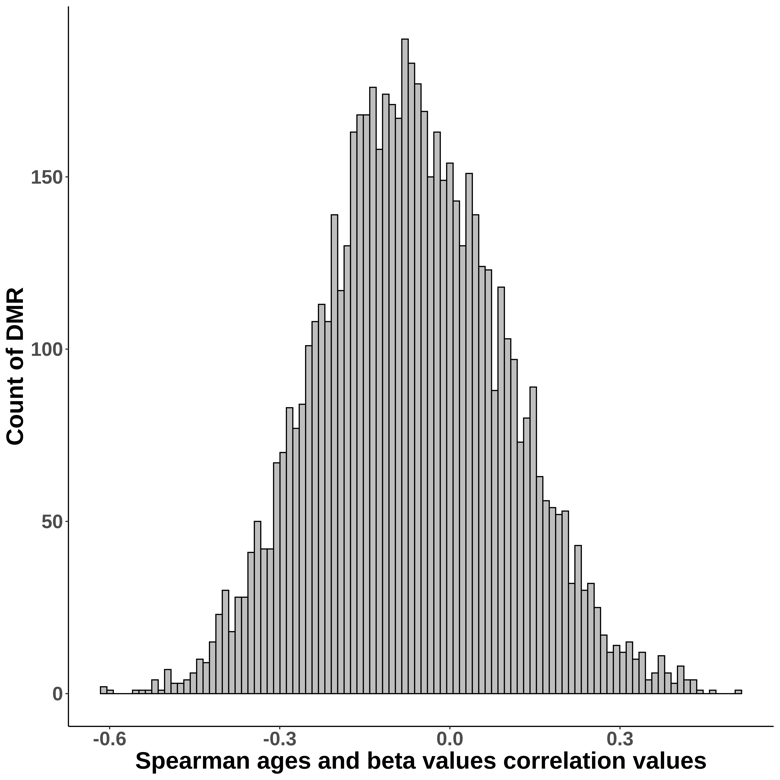


**Supplementary figure 7**: Histogram showing the distribution of correlation values for the association between DMRs beta values and healthy donors ages (n = 54 healthy samples).


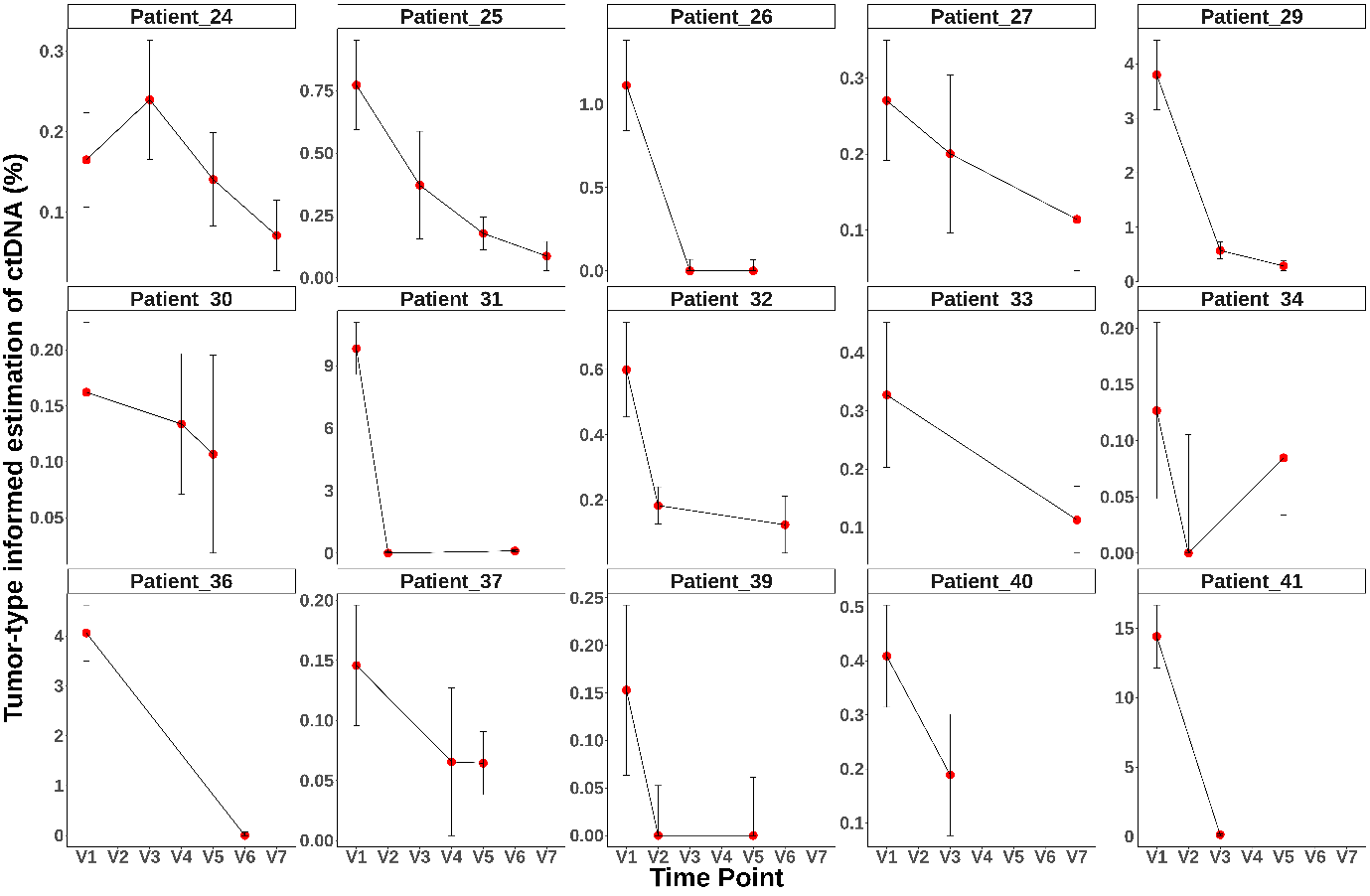


**Supplementary figure 8**: ctDNA follow-up with the tumor-type informed approach for patients with multiple plasma samples available and not analyzed with tumor-informed approach. Error bars show the 95% confidence interval (CI95) of beta values for DMRs selected for ctDNA quantification. Red points indicate positive predicted value for ctDNA presence, while black points are negative.

|  | **Relapse** |  | **No relapse** | **Total** |
| --- | --- | --- | --- | --- |
| **CA125 normalization** | **8** |  | **5** | **13** |
| **No CA125 normalization** | **2** |  | **0** | **2** |
| **Total** | **10** |  | **5** | **15** |

**Supplementary table 1.** Contingency table comparing CA125 normalization status at the end of treatment and relapse occurrence. Among the 15 patients with available data, 9 experienced relapse and 6 did not. CA125 normalization was observed in 13 patients, while 2 patients showed no normalization. Fisher’s exact test revealed no significant association between CA125 normalization and relapse (odds ratio = 0.0, *p* = 0.524).

|  | **Relapse** | **No relapse** | **Total** |
| --- | --- | --- | --- |
| **Cytoreduction score: CC0** | **7** | **6** | **13** |
| **Cytoreduction score:**  **CC1/CC2/NA** | **5** | **1** | **6** |
| **Total** | **12** | **7** | **19** |

**Supplementary table 2.** Contingency table showing the association between cytoreduction score at interval cytoreductive surgery and relapse occurrence. Among the 19 patients included, 13 achieved complete cytoreduction (CC0), and 6 had either suboptimal cytoreduction (CC1/CC2) or did not undergo surgery (NA). Fisher’s exact test indicated no significant association between cytoreduction status and relapse (odds ratio = 0.23, *p* = 0.33).

|  | **Relapse** | **No relapse** | **Total** |
| --- | --- | --- | --- |
| **Absence of lesions on EoT CT** | **6** | **7** | **13** |
| **Presence of lesions on EoT CT** | **6** | **0** | **6** |
| **Total** | **12** | **7** | **19** |

**Supplementary table 3.** Contingency table showing the association between the presence or absence of cancerous lesions assessed by computed tomography (CT) and relapse occurrence. Among the 19 patients included, 13 showed no detectable lesions at the end of treatment (EoT), while 6 had persistent lesions. Fisher’s exact test indicated significant association between CT conclusion status and relapse (odds ratio = 0, *p* = 0.044).

|  | **ctDNA-positive** | **ctDNA-negative** | **Total** |
| --- | --- | --- | --- |
| **Presence of lesions on EoT CT** | **5** | **0** | **5** |
| **Absence of lesions on EoT CT** | **6** | **1** | **7** |
| **Total** | **11** | **1** | **12** |

**Supplementary table 4.** Contingency table showing the association between the presence or absence of cancerous lesions assessed by end of treatment (EoT) computed tomography (CT) and ctDNA detection using the tumor-type informed approach. Among the 12 who relapse and had a CT scan at the EoT, the tumor-type informed approach demonstrated improved sensitivity in predicting relapse compared to CT scan (McNemar’s test p = 0.04).
